# Whole Cells of Microorganisms—A Powerful Bioanalytical Tool for Measuring Integral Parameters of Pollution: A Review

**DOI:** 10.3390/bios15050290

**Published:** 2025-05-04

**Authors:** Maxim Cheliukanov, George Gurkin, Roman Perchikov, Anastasia Medvedeva, Tatyana Lavrova, Tatyana Belousova, Aleksandra Titova, Yulia Plekhanova, Sergei Tarasov, Anna Kharkova, Vyacheslav Arlyapov, Philippe Mandin, Hideaki Nakamura, Anatoly Reshetilov

**Affiliations:** 1 Federal State Budgetary Educational Institution of Higher Education, Tula State University, Tula 300012, Russia; m.s.chelyukanov@tsu.tula.ru (M.C.); g.k.gurkin@tsu.tula.ru (G.G.); r.n.perchikov@tsu.tula.ru (R.P.); ilyuhina.nastya@mail.ru (A.M.); lavrova0000@yandex.ru (T.L.); t.s.belousova@tsu.tula.ru (T.B.); a.s.titova@tsu.tula.ru (A.T.); anyuta_zaytseva@mail.ru (A.K.); 2 Federal Research Center (Pushchino Scientific Center for Biological Research of the Russian Academy of Sciences), G.K. Skryabin Institute of Biochemistry and Physiology of Microorganisms, Russian Academy of Sciences, Pushchino 142290, Russia; yu_plekhanova@pbcras.ru (Y.P.); setar25@gmail.com (S.T.); 3 IRDL UMR CNRS 6027, Université de Bretagne Sud, 56100 Lorient, France; philippe.mandin@univ-ubs.fr; 4 Department of Liberal Arts, Tokyo University of Technology, 1404-1 Katakura, Hachioji 192-0982, Tokyo, Japan; nakamurahd@stf.teu.ac.jp

**Keywords:** microbial biosensor, Internet of Things, artificial intelligence, electroactive biofilms, biological oxygen demand, toxicity, heavy metals, phenol, surfactants, pesticides

## Abstract

Microbial biosensors are bioanalytical devices that can measure the toxicity of pollutants or detect specific substances. This is the greatest advantage of microbial biosensors which use whole cells of microorganisms as powerful tools for measuring integral parameters of environmental pollution. This review explores the core principles of microbial biosensors including biofuel devices, emphasizing their capacity to evaluate biochemical oxygen demand (BOD), toxicity, heavy metals, surfactants, phenols, pesticides, inorganic pollutants, and microbiological contamination. However, practical challenges, such as sensitivity to environmental factors like pH, salinity, and the presence of competing substances, continue to hinder their broader application and long-term stability. The performance of these biosensors is closely tied to both technological advancement and the scientific understanding of biological systems, which influence data interpretation and device optimization. The review further examines cutting-edge developments, including the integration of electroactive biofilms with nanomaterials, molecular biology techniques, and artificial intelligence, all of which significantly enhance biosensor functionality and analytical accuracy. Commercial implementations and improvement strategies are also discussed, providing a comprehensive overview of the state-of-the-art in this field. Overall, this work consolidates recent progress and identifies both the potential and limitations of microbial biosensors, offering valuable insights into their future development for environmental monitoring.

## 1. Introduction

**Figure 1:**
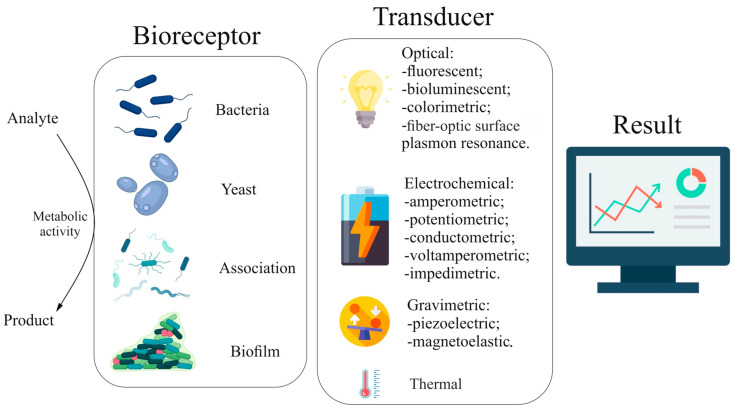
The main types of microbial biosensors.

Biosensors are devices that use biological components (bioreceptors) to detect and quantify various substances. They are widely used for environmental monitoring [1,2,3], food quality [4], agronomy [5], and medicine [6]. Microbial biosensors function due to the metabolic activity of microorganisms, which changes in response to the presence of the analyte, which is recorded by the transducer as an analytical signal. Individual strains of mono-culture microorganisms (bacterial and yeast biosensors), artificially formed associations of microorganisms, and natural communities (activated sludge) are used as biomaterial in biosensors based on whole cells. According to the methods of formation, it can be divided into two large groups—biofilms and cell suspension. Depending on the type of transducer used, biosensors can be divided into several groups: electrochemical, optical, thermal, and gravimetric (Figure 1).

**Figure 2:**
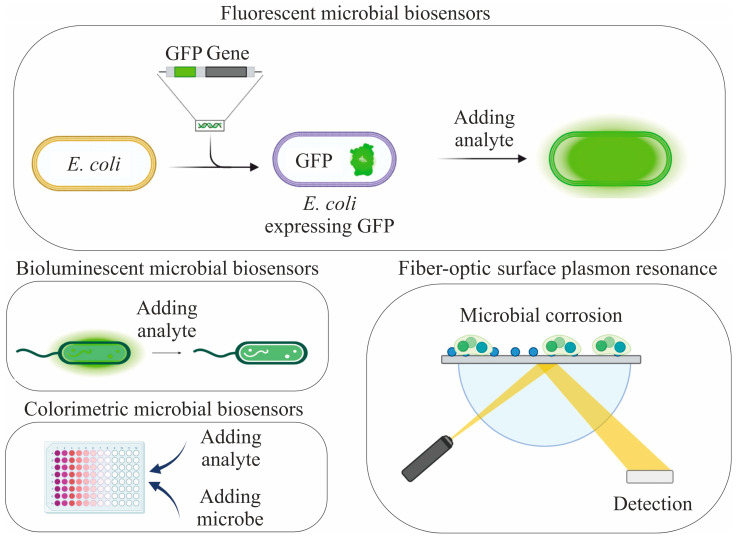
Approaches for the formation of optical microbial biosensors.

An optical transducer is used to change optical parameters such as adsorption, fluorescence, luminescence, or refractive index, which are caused in response to the metabolic activity of microorganisms in the presence of detectable substances in the sample [7]. The main approaches to the formation of optical microbial biosensors are shown in Figure 2.

Fluorescent microbial biosensors are based on the use of genetically modified microorganisms capable of producing fluorescent proteins in response to the presence of certain substances. A construct consisting of an inducible promoter and a reporter gene encoding the synthesis of a fluorescent protein is embedded in the genome of microorganisms. In the presence of a target analyte that interacts with the promoter it is activated, which, in turn, triggers the synthesis of a fluorescent protein. This protein emits light, the intensity of which is directly proportional to the concentration of the analyte. A recent study described a biosensor based on *E. coli* bacteria containing the *luxI*/*IuxR* genes for quantitative lysine monitoring. This biosensor demonstrated high specificity with respect to other amino acids, and its lysine detection limit was only 256 nM [8]. Bioluminescent biosensors are devices that use natural bioluminescence processes observed in living organisms. Bioluminescence is based on a reaction between luciferin and the enzyme luciferase. In the presence of oxygen and cofactors such as ATP, luciferin is oxidized, which leads to the release of energy in the form of light. The review by the authors of [9] describes in detail the main areas of application of bioluminescent microbial biosensors. Colorimetric microbial biosensors work on the principle of measuring the color of the analyzed solution during the interaction of microorganisms with certain chemicals. The review by the authors of [10] contains information about a variety of microbial biotests that allow rapid diagnostics without the use of additional detectors. A recent study [11] presented a device for detecting coronavirus. This device used genetically modified yeast capable of binding the viral protein SARS-CoV-2. Gold nanoparticles functionalized with horseradish peroxidase and antibodies became the second important component for detecting coronavirus proteins. When SARS-CoV-2 spike proteins are present in the system, it forms a “sandwich” that allows for efficient binding and concentration with target molecules. In the presence of SARS-CoV-2 proteins, peroxidase initiates a reaction with tetramethylbenzidine, which leads to a change in the color of the solution. Fiber-optic detectors are also widely used for biofilm research. Their basic principle of operation is to register changes in the refractive index of the medium surrounding the sensor element. For example, in a study [12] a fiber-optic sensor with a ball resonator was developed to monitor the growth of *Pseudomonas aeruginosa* biofilm. A recent study has developed a fiber-optic device capable of detecting microbial corrosion on oceanic objects [13]. This sensor is a surface plasmon resonance device that has been modified by the C-terminus of the BrlR protein. This protein is a specific receptor for the pyocyanin molecule produced by the bacteria *Pseudomonas aeruginosa*, which leads to corrosion. Optical biosensors are small, affordable, highly responsive, and discriminating. However, they are susceptible to external factors and physical changes [14].

**Figure 3:**
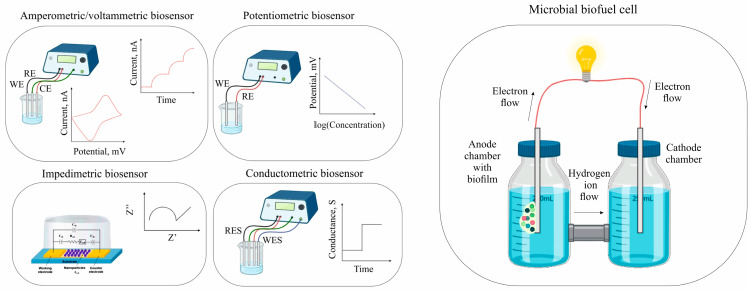
Approaches for the formation of electrochemical microbial biosensors.

To form electrochemical biosensors, bacteria, yeast, or mammalian cells are selected based on their ability to interact with the target analyte, and then the microorganisms are immobilized on the electrode surface, which can be achieved using various methods such as physical adsorption, covalent binding, or encapsulation in a polymer matrix. The choice of the immobilization method is crucial because it affects cell viability and the overall sensitivity of the biosensor. When an analyte binds to a cell or is metabolized by it a biochemical reaction occurs leading to the formation of electroactive particles or a change in the metabolic state of the cell, which is controlled by electroactive particles and which is recorded by various electrochemical methods such as amperometry [15], voltammetry [16], potentiometry [17], conductometry [18], electrochemical impedance spectroscopy (EIS) [19], and microbial fuel cells [20] (Figure 3). The electrochemical sensors in biosensors offer excellent monitoring limits, capabilities, adaptability, and ease of modification and production. They also allow for faster detection, consistent output, and superior resolution. Researchers aim to improve long-term stability, providing steady voltage and current to broad and limited temperature ranges, as well as to solve the problem of hypersensitivity to other components in samples [21].

Gravimetric microbial biosensors are based on the measurement of mass changes related to the metabolic activity of microorganisms. When interacting with target substances, microorganisms begin an active metabolism which leads to the formation of new products, changes in cell concentration, and the release or absorption of water. All these processes can lead to a change in the mass of the system. A gravimetric sensor, usually based on resonance principles (for example, in the form of microbalances), measures mass changes using technologies such as quartz microbalances, which change their resonance frequency depending on the mass attached to their surface. Changes in the resonance frequency are associated with changes in mass, which makes it possible to quantify the activity of microorganisms and, consequently, the concentration of target substances. Gravimetric microbial biosensors are affordable and straightforward, but their sensitivity is limited. Piezoelectric biosensors use piezoelectric materials (such as quartz) that generate an electric charge in response to mechanical stress. Mass changes associated with biochemical reactions or the adsorption of molecules on the sensor surface lead to a change in the oscillation frequency of the piezoelectric element [22,23]. Piezoelectric biosensors are known for their rapid detection, strong frequency response, compact size, and high sensitivity. However, they have limitations, such as their sensitivity to extreme temperatures and their inability to operate in static environments. For gravimetric measurements, magneto-elastic materials can be used, which change their mechanical properties, including stiffness and elasticity, in response to changes in the external magnetic field. Ferromagnetic alloys are one example of such materials. Changes in mass associated with metabolic activity lead to changes in the mechanical properties of the magnetoelastic material. This change can be detected by changing the resonant frequency of a magnetically elastic material [24].

Thermal microbial biosensors are based on measuring temperature changes that occur as a result of the metabolic activity of microorganisms. The biosensor is equipped with a thermal sensor that can accurately measure temperature changes as a result of the metabolic activity of microorganisms, for example, using thermocouples, thermistors, or other types of temperature sensors [25]. Thermal biosensors are versatile, user-friendly, and simple to produce. However, they have limitations in terms of their ability to accurately measure temperature and the time-consuming nature of the experimental process.

**Figure 4:**
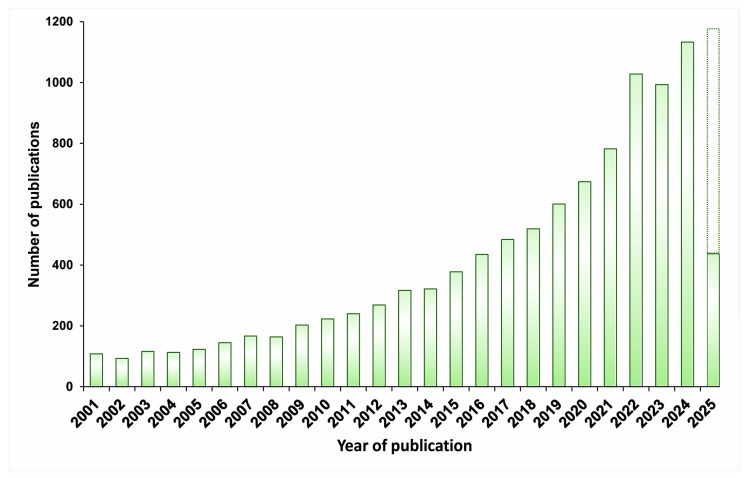
The dynamics of publications on the topic “Microbial biosensor” according to the ScienceDirect database (the dotted line marks the expected growth of publications).

In the last five years, there has been an active growth in publications related to microbial biosensors (Figure 4). One possible factor is the emergence of new technologies.

This review explores the latest developments in microbial biosensors, including the integration of electroactive biofilms with nanomaterials and the application of molecular biology techniques. The use of artificial intelligence to enhance the performance and precision of these sensors is also explored. The review also discusses the commercialization and improvement strategies, providing a comprehensive overview of current trends in this field. It is worth noting that the review examined biosensors for the analysis of a variety of pollutants and indicators, including biochemical oxygen demand, heavy metals, toxicity, surfactants, phenols, pesticides, and microbial contaminations. In summary, the review provides a summary of the latest advancements in microbial biosensors, highlighting their capabilities and limitations and offering valuable insights for future environmental monitoring.

## 2. Approaches to Improving Characteristics of Bioanalytical Systems Based on Whole Microbial Cells

The development of bioanalytical systems using whole microbial cells has attracted considerable attention because of the inherent advantages these systems offer in terms of sensitivity, specificity, and the ability to operate in challenging environments. Various approaches were explored to improve the performance of these systems, with a focus on optimizing microbiological activity, improving detection methods, and integrating advanced technologies.

One of the main strategies for improving bioanalytical systems based on whole microbial cells includes the improvement of bioelectrochemical systems (BESs), which include the previously mentioned MFCs. These systems use the metabolic processes of microorganisms to convert chemical energy into electrical energy, which can be used for a variety of applications, including wastewater treatment and environmental remediation [26]. It has been shown that the integration of exoelectrogens, such as *Pseudomonas* species, significantly increases the efficiency of these systems by facilitating electron transfer during microbial metabolism [27,28]. The configuration and design of MFCs can also be optimized to improve their performance, as evidenced by studies examining laminar flow characteristics and their effect on microbiological activity [26]. In addition to optimizing microbial components, advanced materials and technologies play a crucial role in improving the performance of bioanalytical systems. For example, it has been shown that the development of nanocomposite anodes, such as the graphene/polyaniline nanocomposite obtained from cellulose, improves energy production and bioremediation capabilities in benthic microbial fuel cells [29]. In addition, the use of microfluidic technologies allows for the precise control of environmental conditions, ensuring simultaneous cultivation and analysis of microbial cells. It has been shown that this method allows for highly accurate monitoring of metabolic processes and physiological reactions in microbial communities in real time at the level of individual cells [30].

Raman microspectroscopy has also become a powerful tool for expanding the analytical capabilities of microbial bioanalytical systems. Raman spectroscopy allows for the non-destructive analysis of microbial cells, allowing the identification of metabolic states and interactions within microbial communities. The use of stable isotope sensing in combination with Raman microspectroscopy has further expanded the possibilities of the functional analysis of representatives of the microbial community, providing insight into their role and contribution to complex ecosystems [31]. Modern works describe a strategy for miniaturizing Raman spectrometers based on unstable laser diodes, dense packaging of optics and small sensors, but this method still has a disadvantage in terms of its high cost and the limitation of the laser wavelength used [32].

Moreover, eliminating the heterogeneity of microbial populations is important for improving the reliability and accuracy of bioanalytical systems. Studies have shown that understanding the dynamics and fluctuations of microbial physiology can significantly affect the performance of a bioreactor and the overall effectiveness of bioanalytical applications [33]. Using advanced modeling techniques such as Gaussian mixture modeling, researchers can quantify changes in the structure and dynamics of microbial communities, thereby increasing the interpretability of flow cytometry data and other multidimensional datasets [34].

The application of quality by design (QbD) principles in the development of bioanalytical methods is also gaining popularity, allowing a systematic approach to the optimization and validation of methods. This approach emphasizes the importance of understanding the critical variables of the method and their impact on analytical characteristics, which ultimately leads to the creation of more reliable bioanalytical systems [35]. By applying the principles of QbD researchers can ensure that bioanalytical methods are not only effective but also comply with regulatory standards, which facilitates their use in clinical and environmental settings [36].

Two technologies that can contribute to the development of microbial biosensors should be noted: the introduction of artificial intelligence (AI) and Internet of Things (IoTs) technologies. Artificial intelligence technologies are already actively used in various works, as shown in the review by the authors of [37]. Automated AI-based laboratory platforms improve the accuracy and reproducibility of experiments. Spectroscopy and analytical methods using AI accelerate the interpretation of data and the development of new methods. However, a limitation can be the use of a small sample of data, which may cause inaccuracies in the work of artificial intelligence. In this regard, it is necessary to provide as much data as possible in order to bring the AI’s work as close to real conditions. Our recent work demonstrated the possibility of automating the microbial biosensor calibration process using AI, which greatly simplifies and accelerates analysis [38]. In addition, AI can process and analyze large amounts of data obtained from microbial biosensors; therefore, using machine learning methods, it is possible to optimize the design and materials of microbial biosensors. This will increase their sensitivity and selectivity [39]. The introduction of IoTs technology allows remote monitoring using microbial biosensors [40,41]. IoTs technology makes it possible to automate the collection and analysis of data in laboratories, ensuring security and control. The integration of IoTs with AI allows for the real-time analysis of large amounts of data and the diagnosis of possible problems, which will increase the reliability of microbial biosensors. Thus, the integration of AI and IoTs in the development of microbial biosensors can lead to more efficient, accurate, and versatile tools for environmental monitoring, medical diagnostics, and other fields.

One of the important problems when analyzing real objects is the presence of interfering components that can distort the result. It is necessary to take these influences into account in order to obtain reliable analysis results. To do this, researchers use several methods. The first method is to measure the biosensor parameters in the presence of interfering components (heavy metals, toxicants, etc.) in order to assess their effect on the sensor response. During testing, the “introduced-found” method is used, where the substance to be determined is introduced, for example, into a sample of wastewater containing other contaminants [42,43,44,45]. It is important to conduct testing in samples of various origins (wastewater, tap water, natural waters, etc.) in order to take into account all possible interfering influences and compare the result with traditional methods of analysis. The second method involves the use of AI. In [46], this approach was used to eliminate the effect of interfering components on blood glucose analysis by an enzyme biosensor. This method is also promising for microbial biosensors, where the number of potentially interfering components increases significantly due to the use of microorganisms.

In conclusion, the improvement of bioanalytical systems based on whole microbial cells is a multifaceted process that includes optimizing microbial characteristics, integrating advanced materials and technologies, and applying rigorous analytical methodologies. Using the unique properties of microorganisms and applying innovative approaches, researchers can develop bioanalytical systems that are not only effective at detecting and quantifying target analytes but are also capable of operating in complex and dynamic environments. Further study of these strategies will undoubtedly lead to significant advances in bioanalysis with far-reaching implications for environmental monitoring, clinical diagnostics, and biotechnological applications.

## 3. Determination of Pollutants

### 3.1. BOD

Due to the constant growth of the population and the development of industry, the amount of organic substances entering reservoirs is constantly increasing [47]. Excessive intake of organic carbon leads to the destruction of the ecosystem of a reservoir and makes the water unsuitable for drinking water supply and recreation [48]. The biochemical oxygen demand (BOD) is one of the most important parameters of natural water quality. Aerobic microorganisms, oxidizing organic substances, consume oxygen, therefore, the generally accepted method of estimating BOD is to calculate the amount of oxygen consumed for biochemical reactions over a certain period of time (5, 7, or more days) at 20 ± 1 °C and in total darkness. However, the results obtained in different laboratories using this 5-day test may differ by more than 20% due to the use of different species compositions of microbial communities and the complexity of the experiment [49]. In addition, this method requires a lot of time and trained laboratory staff, and it is not suitable for automation in real time. In addition, the use of this method is not limited to the analysis of natural waters, it is also necessary to monitor the operation of wastewater treatment plants and to assess the quality of discharged water, according to standards presented in [50]. Therefore, all the listed problems and challenges make the development of new approaches and methods for the rapid determination of BOD relevant. One of the popular alternative methods of measuring BOD has become the biosensor method. Although more than 40 years have passed since the first report on a BOD biosensor [51], modern researchers still continue to develop new devices, achieving impressive results [52,53,54,55,56,57,58] which we present in this review.

**Figure 5:**
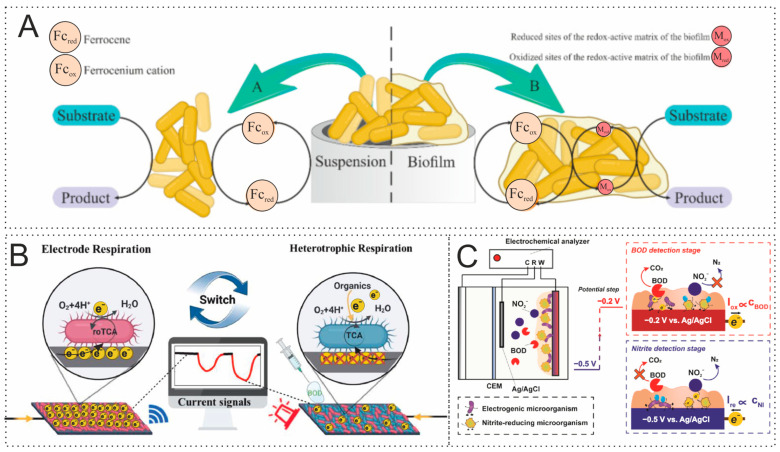
(**A**) The mechanism of electron transfer in the “ferrocene–immobilized microorganisms” system and in the “ferrocene–microbial biofilm” system. Reprinted from ref. [67] Copyright © 2024 Creative Commons Attribution 4.0 License. (**B**) Switching between electrotrophic and heterotrophic respiration makes it possible to sensitively detect BOD, demonstrating the possibility of using bacteria switching between two competing metabolic pathways as biosensors. Reprinted with permission from ref. [64] © 2023 Elsevier B.V. All rights reserved. (**C**) A new two-functional electrochemical biosensor capable of performing the rapid and simultaneous detection of nitrites and dissolved BOD. Reprinted with permission from ref. [70] © 2023 Elsevier B.V. All rights reserved.

To develop biosensors for the rapid determination of BOD, it is necessary to use microorganisms with broad substrate specificity that are capable of oxidizing the maximum number of organic compounds present in water [59]. As a rule, single strains of identified microorganisms [60], a mixture of several strains (association) [61], or activated sludge [62] can be used. Nevertheless, the selection of microorganisms must be carried out based on the objectives of the experiment. The analysis of scientific literature has recently shown an increase in experimental work using electroactive microorganisms (EAMs) and electroactive biofilms (EABs) [62,63,64,65,66]. In general, the effectiveness of using EABs in the design of BOD sensors was studied in [67]. It has been proven that the EABs of bacteria *Ps. veronii*, *E. coli*, and *S. cerevisiae* yeast represent an excellent alternative to cells of microorganisms that do not possess electroactivity. Such systems are capable of measuring BOD for very pure water (BOD less than 1 mg/L) in a short time of 5–10 min with a high correlation to the standard BOD determination method. Moreover, electron transfer in such systems proceeds much better due to mediators synthesized by bacteria in the biofilm (Figure 5A). Such results indicate a great prospect for using EABs for the rapid determination of BOD, although such systems have a long startup time due to the long development of biofilms. In addition to the problem of monitoring natural freshwater for the BOD indicator, there is also a problem in the analysis of seawater, which consists of high salinity. Due to the artificial adaptation of microorganisms, the authors of [68] managed to overcome this barrier and develop a simple strategy for preparing a sensor with a biofilm reactor for BOD detection. The developed method made it possible to universally determine the BOD of both freshwater and seawater, overcoming the limitation that the sensor can only be used in one type of reservoir due to poor adaptation to the environment. In addition to all of the above, the use of EAMs and EABs opens up the possibility of the double determination of BOD and nitrites. The study [69] was the first to propose a method for the simultaneous measurement of BOD and nitrates in water using EAMs due to bidirectional extracellular electron transfer. The authors of [70] also provided a realistic way to develop multifunctional biosensors for detecting pollutants with various redox properties. The authors were able to achieve accurate responses of dissolved BOD in the range of 5–100 mgBOD/L and nitrites in the range of 0.05–16 mgNO_2_/L with a single measurement time of 20 min. These works are interesting because they open up new knowledge about the creation of multisensors capable of rapid monitoring of water by several indicators. The possibility of using facultative electrotrophs as a sensing element has been shown in the research of [64]. This new BOD detection principle allows measurements to be performed by switching between electrotrophic and heterotrophic respiration. The results of the work demonstrated the possibility of using bacteria with two competing respiratory tracts to detect contamination in natural water (Figure 5B).

The work described above makes it clear that EAMs and EABs are a new and effective tool in BOD analysis. They can not only improve the characteristics of already studied and formed systems but also allow the discovery of new measurement and detection methods not previously described in the literature. These advantages make EABs and EAMs new candidates for the development of advanced BOD express sensors in the near future.

Undoubtedly, the use of biomaterial plays a crucial role in the characteristics of express sensors for determining BOD. But for effective operation and better performance, methods of cell immobilization, the use of mediators, and the presence of substances in the bioreceptor element that promote electron transfer are also important. Thus, for the development of BOD biosensors in [71], a method for the formation of receptor systems based on the different stages of bioelectrocatalysis of mediators is used. For example, there is a known practice of using redox-active polymers to improve the properties of biosensor systems [72,73,74,75]. The advantages of the ferrocene mediator and the yeast *Blastobotrys adeninivorans* have been demonstrated. The effectiveness of *B. adeninivorans* and dual-mediator systems was also confirmed in [76]. Electropolymerized thionine was used as a redox-active polymer to create a bi-mediated microbial biosensor for BOD detection. It has been shown that the most promising system in terms of the rate of interaction with yeast is based on poly(thionine), single-walled carbon nanotubes (SWCNTs), and neutral red. Although the presence of a second mediator can increase the cost of the finished structure, such solutions make it possible to measure BOD more efficiently. Therefore, it is necessary to use cheap and affordable mediators in such systems.

To achieve a constant number of microorganisms and the efficient operation of sensors, researchers resort to various methods of immobilization of biomaterial. In work [77], *Paracoccus yeei* VKM B-3302 bacteria were immobilized in a hydrogel matrix. The developed hydrogel forms a mesh structure that allows it to capture microbial cells while preserving their viability and biocatalytic properties, which provides the technological possibility of replicating single-use receptor elements of the biosensor. This work also reports on a very low limit of detectable concentrations—0.05 mg BOD/L—which makes it possible to measure very pure samples. The correlation to the standard method, which was R2 = 0.9990, also indicates the use of this method instead of the generally accepted one with high speed and accuracy.

The analysis of the literature also notes the use of sol–gel chemistry for the immobilization of microorganisms in the design of BOD sensors [78,79,80]. The use of matrices obtained during the sol–gel process makes it possible to achieve excellent long-term stability, as well as neutralize the effect of heavy metals [79].

Microbial fuel cells are an innovative technology that is increasingly being widely used in the field of ecology and water quality analysis, in particular for the determination of BOD [63,81,82,83]. One of the key advantages of MFCs is their ability to provide high sensitivity and specificity in the assessment of organic pollution, which makes them an effective tool for monitoring aquatic ecosystems [84]. In addition, the use of microbial cultures allows for real-time and offline analysis, which significantly reduces the time to obtain results compared to traditional methods [85]. The researchers of [86] succeeded in developing an X-ray microbial fuel cell and applying it to determine the BOD. The manufactured sensor allowed operation with a volume of 1.8 µL, providing excellent response to various concentrations of BOD. At the same time, the cost of this sensor is low and amounts to only 0.5 USD. This paper is an excellent example of using MFCs to determine the BOD. However, despite the obvious advantages such as environmental sustainability and the possibility of reuse, there are some disadvantages, including dependence on the growing conditions of microorganisms and the need for careful calibration of the system to ensure the accuracy of measurements [87]. In [85], the operability of autonomous MFCs for determining BOD at various temperatures was investigated. As a result, it was found that the performance of MFC-based BOD biosensors will be strongly influenced by environmental changes during long-term operation due to the inherent unpredictability of biological processes. The results showed that an increase in operating temperatures from 15 ± 1 °C to 35 ± 1 °C can significantly reduce the startup time of an MFC-based biosensor. Thus, microbial fuel cells represent a very promising tool for determining BOD, which combines both advantages and challenges that require further study and optimization.

**Figure 6:**
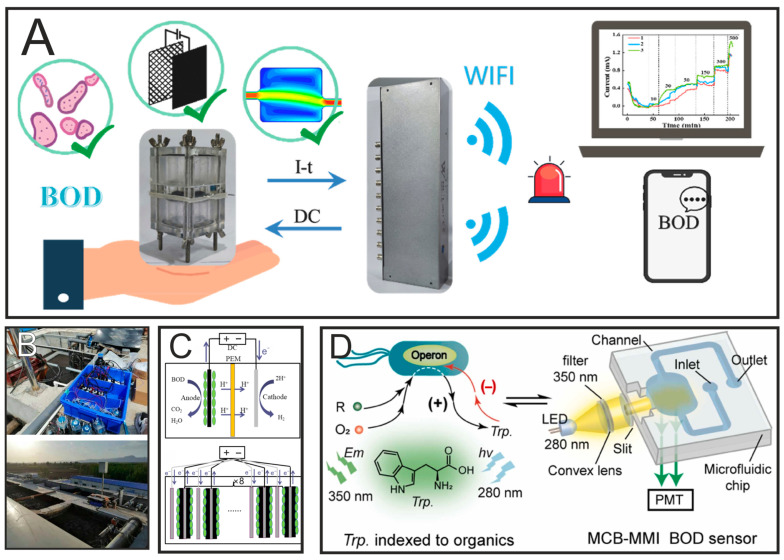
(**A**) Development, optimization, and application of a highly sensitive BOD biosensor based on microbial electrolytic cells [88]. (**B**) Photography and the environment of the BOD online monitoring system installation in real time [88]. (**C**) Schematic diagram of a two-chamber MFC and a multilayer MEC reactor. Reprinted with permission from ref. [88] © 2023 Elsevier B.V. All rights reserved. (**D**) A BOD sensor with a microbial metabolism index based on a microfluidic chip for the rapid determination of biochemical oxygen demand. Reprinted with permission from ref. [89] © 2023 Elsevier B.V. All rights reserved.

Currently, studies of BOD biosensors based on microbial electrolytic cells (MECs) (Figure 6A–C) are at the stage of theoretical research. Therefore, the authors of [88] designed and optimized such a biosensor. The formed BOD biosensor was used to determine the concentration of BOD in the laboratory, where it accurately measured BOD concentrations in the range of 10–500 mg BOD/L and maintained a good correlation to the standard method of determination. Then, BOD sensors such as microbial electrolytic cells were put into practice in the field as alarms in case of accidents at real sewage treatment plants. The formed BOD detection system was quickly assembled on site and launched, and it issued an early warning shortly after a sudden increase in the concentration of organic substances in the water, indicating a high potential for determining environmental hazards.

In addition to using MFCs, it is possible to determine the BOD using optical converters. For example, in [89] the authors proposed a new strategy for rapid in situ BOD detection using a microfluidic chip-based BOD sensor indexed by microbial metabolism. The rapid detection of BOD was achieved by quantifying the equilibrium concentration of tryptophan involved in aerobic biodegradation, alternating with traditional 5-day monitoring of dissolved oxygen changes (Figure 6D). Comparative analysis shows that optical sensors have a faster response when MFCs demonstrate increased stability in long-term monitoring. At the same time, problems may arise with MFC sensors due to the long startup time and changes in the prevailing microorganisms, which can lead to variability in sensor responses. Therefore, in future work, researchers will need to study in detail the implementation of systems in applied industries.

Moving into the modern era, there is a need to introduce artificial intelligence and machine learning to develop devices and improve accuracy [37], including for new-generation BOD sensors. Although at the moment there is a small amount of work devoted to the implementation of machine learning and AI for the development of BOD sensors, the authors of [90] managed to develop a new software sensor using eXtreme Gradient Boosting (XGBoost) machine learning to determine extreme BOD values. The formed sensor was tested on the BOD in sewage treatment plants to confirm the results. Also in [91], an artificial neural network was created that predicts the BOD within 6–24 h with an average error of 7%. The models were trained on the voltage data obtained by MFCs for 24, 16, 12, 8, 6, and 2 h, and were used to predict the BOD values. However, it should be noted that although the use of artificial intelligence and machine learning shows encouraging results in improving the accuracy and efficiency of BOD sensors, there is a need for further research to optimize algorithms and increase their resistance to various operating conditions. In addition, it is necessary to take into account possible limitations and errors associated with the training data, which may affect the accuracy of forecasts.

### 3.2. Toxicity

**Figure 7:**
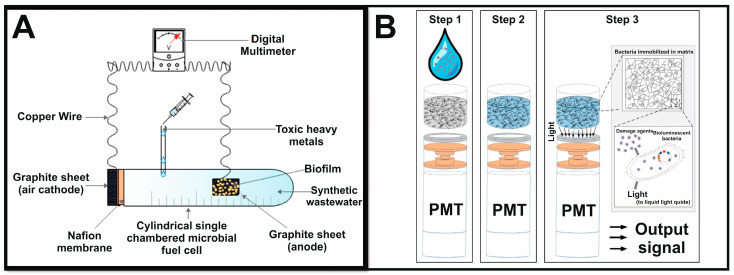
(**A**) Schematic representation of a cylindrical single-chamber biosensor based on an MFC. Reprinted with permission from ref. [94] © 2021 Elsevier Ltd. All rights reserved; (**B**) schematic representation of the biosensor and the measurement process: step 1. adding a water sample, step 2. diffusion of toxic substances into the matrix, step 3. toxic substances cause a bacterial reaction. Reprinted with permission from ref. [95] Copyright © 2015 Elsevier B.V. All rights reserved.

Microbial biosensors represent a significant advancement in environmental monitoring, especially in the detection of toxic substances such as heavy metals and organic pollutants [92]. These biosensors use biological recognition elements in combination with transduction systems to transform biochemical interactions into measurable electrical signals, facilitating real-time monitoring and assessment of pollutants in environmental, agricultural, and biomedical contexts. Biotesting using various living organisms, from bacteria and cell cultures to fish and crustaceans (daphnia), is used to assess the toxicity of media [93]. However, such methods are often time consuming. As a quick, economical and simple alternative, the use of microorganisms is considered, whose reaction to toxicants is similar to that of more complex organisms. This significantly reduces the time and cost of analysis. The integration of microbial cells into biosensors allows for the autonomous monitoring of environmental conditions in real time, which is crucial for the timely control of toxicants (Figure 7A) [94].

The use of genetically engineered microorganisms in biosensors is gaining momentum due to their increased sensitivity and specificity for detecting specific pollutants. For example, researchers have successfully developed microbial biosensors by combining luminescent genes such as *lux*, *gfp*, or *lacZ* with gene promoters that respond to the presence of heavy metals in water. This approach makes it possible to determine the bioavailability and toxicity of these metals, which makes it a powerful tool for environmental quality management [96]. In addition, microbial biosensors can be used to create and optimize a new biosensor that combines the following two systems: non-consumable (optoelectronic devices) and consumable (bioluminescent bacteria immobilized in a calcium alginate matrix). Ease of maintenance and measurement, as well as portability and sensitivity, make this device attractive for use in water quality analysis and environmental monitoring (Figure 7B) [95].

It has also been shown that the integration of MFC technology into biosensor applications facilitates the real-time monitoring of toxicity as the metabolic activity of microorganisms is used to generate electrical signals that correlate with the concentration of toxicants [97,98].

The development of mediator microbial biosensors has also been studied as a means of increasing the sensitivity and specificity of toxicity detection in surface waters [98,99]. These systems use stable microbial associations that are sensitive to various toxins, including heavy metals and organic substances. As research has progressed, electrochemical microbial biosensors have become one of the most widely used platforms due to their high accuracy and versatility in detecting various target materials, including glucose, heavy metals, and phenolic compounds. Thus, in [100], a highly sensitive biosensor based on the bacterium *Paracoccus yeei* and a ferrocene mediator was developed to determine the toxicity of samples of perfumery, cosmetics, and wastewater. In the course of the study, the optimal mediator of electron transfer was selected based on the analysis of the constants of interaction with the biomaterial and the efficiency of electron transfer. Ferrocene immobilized in graphite paste showed the best results. Testing of the biosensor on samples of heavy metals and phenols confirmed its high sensitivity to toxicants and its promise for real-time monitoring. Although covalently bound mediators, for example, in the BSA–NR–CNT system, exhibit a high interaction constant with *P. yeei*, low electron transfer rates can limit sensitivity. Biological methods for assessing toxicity, in particular, electrochemical microbial biosensors, are advantageous due to their simplicity, speed of analysis, low cost, and the possibility of miniaturization. The luminescent type of toxicity detection, although effective, creates problems when choosing the biomaterial used, which prompted researchers to focus on electrochemical methods to increase efficiency [101]. In addition, the use of whole-cell fluorescent biosensors has been noted as a promising approach for assessing the bioavailability and biodegradation of complex pollutants such as polychlorinated biphenyls [102].

**Figure 8:**
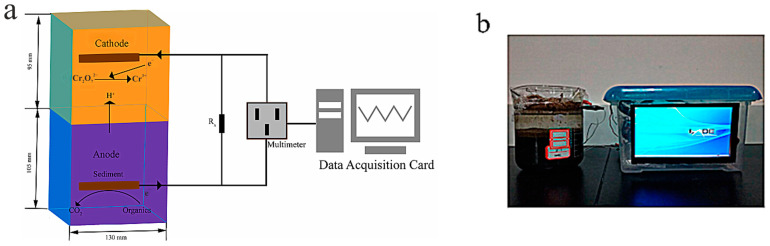
A block diagram of the biosensor’s design and operation. (**a**) Schematic representation of a microbial fuel cell biosensor in 3D; (**b**) biosensor operation interface. Reprinted from ref. [104] © 2018 by the authors.

The versatility of microbial biosensors is confirmed by their use to detect specific environmental pollutants such as mercury and cadmium. These biosensors emit quantifiable signals in response to intracellular concentrations of these metals, providing valuable information about their bioavailability and potential toxicity [103]. Moreover, advances in biosensor technologies have made it possible to develop portable and cost-effective systems that can be deployed on-site for the real-time monitoring of industrial wastewater, in particular, for detecting pollutants such as hexavalent chromium in the cathode space (Figure 8) [104].

In the context of heavy metal detection, the response of microbial biosensors is often attributed to soluble ionic forms of metals that can be easily absorbed by microbial cells. Recent studies have shown that the bioavailability of metals can be influenced by their chemical forms, which requires the development of biosensors that can distinguish between these forms to provide accurate estimates of toxicity [105]. The inclusion of nanomaterials in biosensor designs has also been studied to increase sensitivity and detection limits, especially for trace levels of toxicants [106].

The use of microbial biosensors goes beyond environmental monitoring. They are also used in food safety and quality assessment. For example, biosensors have been developed to detect pesticide residues and other pollutants in food, ensure compliance with safety regulations, and protect public health [107]. The ability of microbial biosensors to provide fast and reliable results makes them invaluable tools both in the context of environmental safety and in the field of food safety.

Thus, microbial biosensors represent an advanced approach to toxicity detection that uses the natural capabilities of microorganisms to effectively monitor environmental pollutants. Their application covers a wide range of areas, including environmental monitoring, food safety, and industrial wastewater management. As research in this field continues to evolve, the integration of synthetic biology and nanotechnology into biosensor design has the potential to further enhance the sensitivity, specificity, and applicability of these innovative tools.

### 3.3. Heavy Metals

Along with the determination of integral toxicity, it is important to identify individual toxicants. Heavy metals such as lead, cadmium, mercury, and arsenic pose a serious threat to the environment and human health. Their presence in water, soil, and food products can cause various diseases [108,109,110,111]. In the human body, heavy metals accumulate in such important organs of the human body as the brain, heart, liver, and kidneys, thereby destroying normal biological functions [112,113]. Heavy metals can bind to proteins, including enzymes, and change their three-dimensional structure, which leads to its deactivation or the disruption of its functioning [114]. A study [115] shows that an increased content of cadmium in the blood is associated with an increase in mortality from heart and vascular diseases, and a high level of lead increases the risk of death from various types of cancer. Table 1 shows the MPCs of heavy metals listed in Chinese and European regulatory documents [116].

Traditional methods for the determination of heavy metals, such as atomic absorption spectroscopy (AAS) and optical emission spectroscopy (OES), require sophisticated equipment and long analysis time [117,118]. While these methods have the advantage of being able to simultaneously measure the multiple analytes qualitatively and quantitatively, they have the disadvantage of being unable to measure the toxicity to living organisms. In contrast, biosensors based on the use of whole cells of microorganisms represent a promising alternative to traditional methods of analysis. This is because various biosensors that use whole cells of microorganisms are being studied, and some types can quantitatively measure analytes, while others can measure toxicity. Upon contact with heavy metals in the analyzed sample, microorganisms react by changing their metabolic activity. This change is detected by a sensor and converted into a measurable signal, such as a change in electric current, luminescence, or optical absorption. The analysis of the received signal makes it possible to determine the concentration of heavy metals in the test sample. The use of whole cells of microorganisms in biosensors is usually cheaper than traditional methods of heavy metal analysis since the use of expensive equipment is excluded. Also, biosensors with whole cells can be designed with a simple design, making them available for widespread use. Such devices are highly effective in determining the concentration of heavy metals in various aquatic environments, including drinking water, wastewater, and reservoirs [119,120]. Due to their sensitivity and specificity, biosensors can detect excess levels of heavy metals in a timely manner and prevent negative consequences for human health and ecosystems [121,122]. Biosensors can be used to monitor the level of heavy metal contamination in food and feed [123].

**Figure 9:**
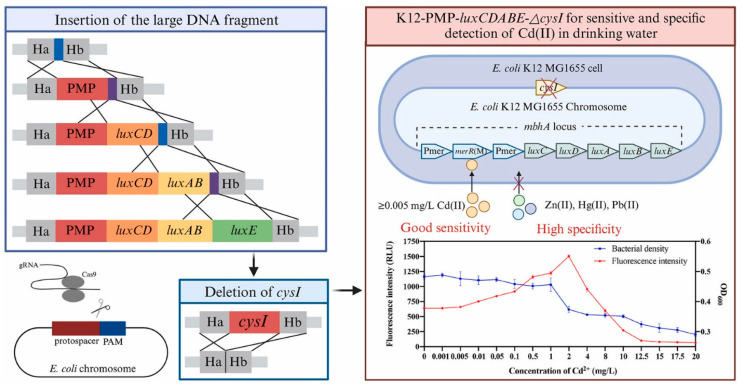
Engineered *E. coli* biosensor for Cd(II) detection using CRISPR/Cas9. Reprinted with permission from ref. [125] © 2024 Elsevier Ltd.

The advantage of using whole microbial cells in biosensors is their high sensitivity to even low concentrations of heavy metals, which makes it possible to detect them at early stages of contamination [119,124]. Some microorganisms exhibit a specific reaction to certain types of heavy metals, making it possible to differentiate their presence in the sample [125,126]. In [125], a biosensor was created by merging the mercury promoter *Pmer*, the regulatory gene *merR*, and the luciferase gene *luxCDABE* into the *E. coli* chromosome using the CRISPR/Cas9 gene editing technology (Figure 9).

Suppression of the *cysI* cadmium resistance gene further increased the biosensor’s sensitivity to Cd^2+^. The designed biosensor demonstrated a good nonlinear response to Cd^2+^ concentrations in the range from 0.005 to 2 mg/L.

**Figure 10:**
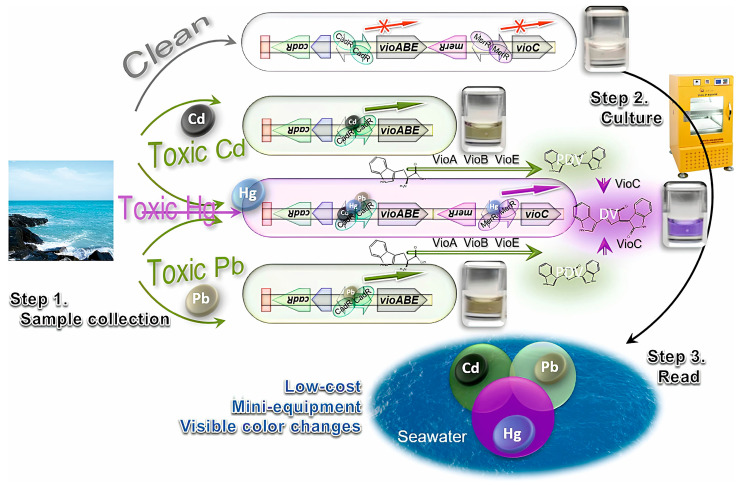
Scheme of action of a two-color bacterial biosensor reacting to cadmium, mercury, and lead to detect the pollution of seawater with heavy metals. Reprinted with permission from ref. [119] © 2024, the authors. Published by Elsevier Ltd.

Such biological sensors allow for fast and accurate analysis, which is especially important for the quality control of agricultural products and ensuring food safety. Due to their portability and ease of use, biosensors prove to be an effective tool for the monitoring environmental pollution and the timely identification of pollution sources. A biosensor based on *Bacillus megaterium* VR1 showed sensitivity to cadmium, copper, and zinc [124]. The lowest detection limit is defined as 1.42 × 10^−4^, 3.16 × 10^−4^, and 2.42 × 10^−4^ mg/L for cadmium, copper, and zinc, respectively. The optimal pH range for the functioning of the developed biosensor is set within 5–8.5. Storage of the biosensor for two weeks did not lead to a significant decrease in fluorescence, demonstrating its high stability. The study in [119] presents a colorimetric two-color bacterial biosensor capable of detecting Cd^2+^, Hg^2+^, and Pb^2+^ in seawater with high sensitivity: 9.7 nM for Cd^2+^, 24.4 nM for Pb^2+^, and 0.5 nM for Hg^2+^ (Figure 10). The biosensor is highly resistant to salinity, and no pretreatment of the sample is required.

In [94], a fuel cell based on microorganisms isolated from sewage treatment plants was able to detect the presence of toxic heavy metal ions Cu^2+^, Cr^6+^, Zn^2+^, and Ni^2+^ in wastewater. The inhibition coefficient of all toxic compounds was calculated based on the respiratory activity of electroactive microorganisms and its inhibition. However, such a system assumes reuse only if the biosensor is restored using a sodium acetate solution within a few days. The recovery time depends on the concentration of the toxicant and its effect on the system. In the event that all the bacteria in the fuel cell are inactivated by toxic shocks without recovery, the biosensors must be replaced. This is a serious problem during in vivo monitoring.

**Figure 11:**
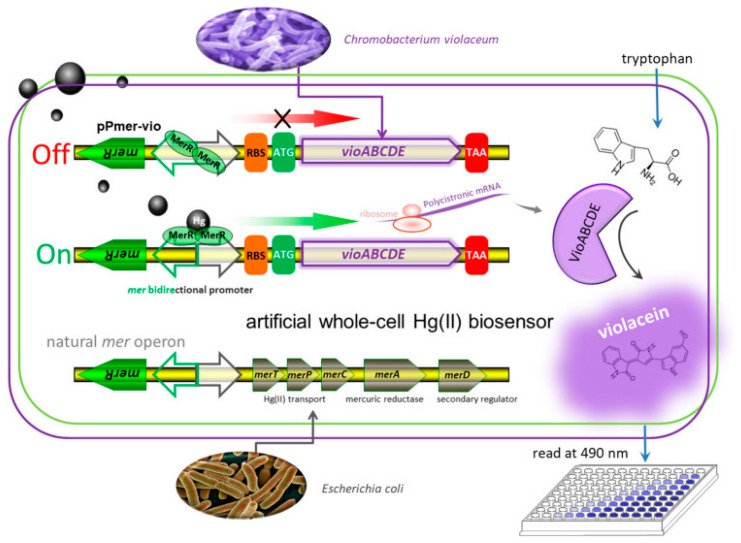
Engineered biosensor Hg^2+^ based on MerR-regulated visual production of violacein. Reprinted from ref. [127] Copyright © 2021, the author(s) Yan Guo et al.

In [127], a new single-cell biosensor was developed for detecting Hg^2+^ in the environment (Figure 11). The biosensor is based on the synthesis of violacein under the control of a mercury promoter and regulator. It has high specificity to Hg^2+^ and it is able to detect low concentrations of Hg^2+^ (0.39 µM for growing cells and 0.006 µM for non-growing ones). The biosynthetic module of violacein, obtained from *Chromobacterium violaceum*, is transcriptionally regulated by the sensitive element Hg^2+^, which makes it possible to create an Hg^2+^ biosensor based on an entire cell. The color change of violacein is easily detectable visually. The biosensor has been successfully validated on real water samples, demonstrating its practical value for monitoring mercury pollution.

In the study [128], a new approach was developed to create microbial biosensors based on paper with biofilm, which makes it possible to reliably fix *Escherichia coli* cells to the surface. The introduction of a cellulose-binding peptide domain into the *CsgA* biofilm protein improves cell adhesion, increasing the efficiency of the biosensor. As a demonstration, a biosensor for copper has been created that can detect its concentrations with a 5 μM limit of detection at clearly visible signal levels even after months of storage. The biosensor is resistant to various environmental conditions and remains functional after freeze-drying and storage for several months. This platform opens up opportunities for the development of affordable, portable, and stable microbial biosensors for a wide range of analytes in the temperature range from 25 °C to 42 °C, at salinity levels from 10 to 100 mM NaCl, and pH values from 7 to 9. However, the authors point out that the problem of maintaining the long-term viability and analytical characteristics of microbial biosensors is still not fully solved, which prevents them from competing with more traditional cell-based assays. The studies [127,128] present sensors designed to detect individual metal ions, as opposed to the complex detection of a combination of heavy metals. For more comprehensive environmental quality monitoring and the assessment of toxic effects, the development of sensors capable of detecting a combination of heavy metals, not just individual ions, is required.

The development of new types of biosensors for the detection of heavy metals is an urgent task in the field of analytical chemistry aimed at increasing sensitivity, specificity, and resistance to external factors. A promising direction is the creation of portable biosensors that allow rapid analysis in the field. The integration of biosensors with other technologies such as microfluidics, nanotechnology, and sensor arrays open up new opportunities for creating multifunctional analytical platforms capable of not only determining the concentration of heavy metals, but also conducting spatial mapping of their distribution, which increases the accuracy and information content of the analysis.

### 3.4. Surfactants

Surfactants are a class of chemicals that have the ability to reduce the surface tension of liquids and increase their detergent properties. The excessive use of surfactants and their uncontrolled release into the environment can cause water pollution. Surfactants pose significant environmental problems due to their widespread industrial use and potential toxicity to aquatic ecosystems. Sodium dodecyl sulfate (SDS) is the most well-known surfactant and a commonly used anionic detergent in household, cosmetics, and commercial substances [129]. It has a variety of applications, for example, as an emulsifier, stabilizer, foaming agent, and cleaning agent [130,131,132]. According to the classification of toxicity, anionic surfactants belong to harmful chemicals [133]. SDS in domestic and industrial wastewater is one of the main causes of pollution of aquatic ecosystems and drinking water. Several studies have reported that SDS has a detrimental effect on the survival and reproduction of organisms in the aquatic ecosystem by interfering with biological processes [134,135,136].

Effective detection of these compounds is crucial for environmental monitoring and remediation efforts [137]. Microbial biosensors are increasingly recognized as valuable tools for this purpose due to several key advantages [138]. Microbial biosensors use living microorganisms as sensitive elements, which makes it possible to detect specific surfactants through their biological reactions. For example, *Herbaspirillum lusitanum* has been shown to effectively detect low concentrations of surfactants, namely sodium dodecyl sulfate, with a detection limit of only 0.01 mg/L, which is below the regulatory thresholds for drinking water [139]. This specificity is crucial for the accurate monitoring of environmental pollutants.

These biosensors not only detect the presence of surfactants but can also assess their biodegradability. Microorganisms used in microbial biosensors can metabolize surfactants, which provides insight into the bioavailability and potential environmental impact of these compounds. For example, the bioluminescence reaction in *Pseudomonas putida* shows how surfactants increase the bioavailability of hydrophobic organic compounds, which is important for understanding their behavior in the environment [140].

Microbial biosensors provide fast analysis time (1–5 min) compared to traditional methods, which makes them suitable for real-time monitoring in various conditions, including wastewater treatment plants [136,139]. Their ability to operate without the need for purified enzymes or complex preparations reduces costs and simplifies field deployment.

These biosensors can be used directly in environmental samples without extensive pretreatment, which allows real-time monitoring of the levels of surfactants in natural reservoirs or industrial wastewater. This opportunity is especially valuable for assessing the effects of surfactants on microbial communities and the general state of the ecosystem [136,139].

Thus, microbial biosensors are vital tools for detecting surfactants due to their specificity, sensitivity, ability to assess biodegradability, rapid analysis capabilities, and suitability for in situ monitoring. These characteristics make them indispensable in environmental monitoring and pollution control strategies. While some strains of microorganisms are resistant, others may not work well under different conditions, which limits their applicability in different environments [141]. Microbial biosensors are also often unable to distinguish between different types of surfactants, which makes them less specific than some chemical methods. This limitation can lead to inaccurate indicators, especially when studying complex environmental samples where several compounds of this class may be present [142,143]. Biological mechanisms inherent in microbial sensors can contribute to these problems, making it difficult to effectively detect various types of surfactants [142].

A notable achievement is the development of microbial biosensors that use engineered bacteria to detect specific surfactants. For example, one study noted a biosensor designed to respond to anionic surfactants, demonstrating effective detection capabilities in an aqueous environment [144,145]. It is worth noting that optimization of the anionic surfactant sensor was studied in [144]. The study showed that at temperatures above 35 °C the response of the formed sensor decreased significantly. The effect of pH was studied in the range from 5.6 to 8.6. The sensor response increased significantly with increasing pH, reaching a maximum in the region of 7. With a further increase in pH, the response gradually decreased. However, this does not take into account the effect of competing organic substances on the sensor response, which may distort the analysis results.

Thus, in [136], the authors present an approach to the creation of a microbial biosensor based on a transcription factor, which is designed for the specific and sensitive detection of SDS. To implement this idea, the researchers selected *Pseudomonas aeruginosa* bacteria, which were modified so that they could serve as the basis for a biosensor. The authors demonstrated that the developed biosensor has high sensitivity, linearity, and an impressive limit, which makes it possible to effectively detect SDS in various environmental samples. One of the key advantages of the developed biosensor is its high specificity to SDS, which allows it to distinguish between two closely related anionic detergents—SDS and SDBS. This biosensor overcomes the limitations of sample processing, complex instrumentation, the use of hazardous chemicals, and the synthesis of specialized polymers or nanomaterials. Nevertheless, the developed biosensor provides remarkable detection of this anionic detergent of extreme importance. The authors believe that the proposed biosensing method has great potential for detecting this xenobiotic in places of medical care and in places where environmental pollution control is critically important. Thus, the work represents an important step forward in the development of biosensors that can be used to monitor pollutants in real time, which in turn can contribute to improving environmental safety.

The study in [145] examines the use of various bacterial strains, in particular from the genera *Pseudomonas* and *Achromobacter*, which are capable of destroying anionic surfactants. The study focuses on the substrate specificity and stability of sensor signals, demonstrating that these microbial biosensors can effectively detect surfactants such as SDS, with a lower detection limit of about 1 µm. This model promises a rapid assessment of surfactants in aquatic environments, especially in wastewater conditions.

In conclusion, the authors concluded that a comparison of sensors based on bacteria of the genera *Pseudomonas* and *Achromobacter* demonstrated significant similarity in terms of substrate specificity, which suggests that they are able to effectively interact with certain substances. Moreover, both bacterial genera have shown high sensitivity to substances such as SDS, sodium alkane sulfate, and some nonionic surfactants. This is important for the development of new analysis methods as sensitivity to these substances allows the use of these sensors for environmental monitoring and water quality control. A microbial biosensor model developed based on these bacteria has demonstrated the ability to detect surfactants with high selectivity and sensitivity. In addition, an important aspect is the reproducibility of the signals, which makes it possible to use this model for a long time without significant changes in its characteristics. This makes microbial biosensors promising tools for analyzing various samples, including wastewater, where the presence of surfactants may indicate contamination.

**Figure 12:**
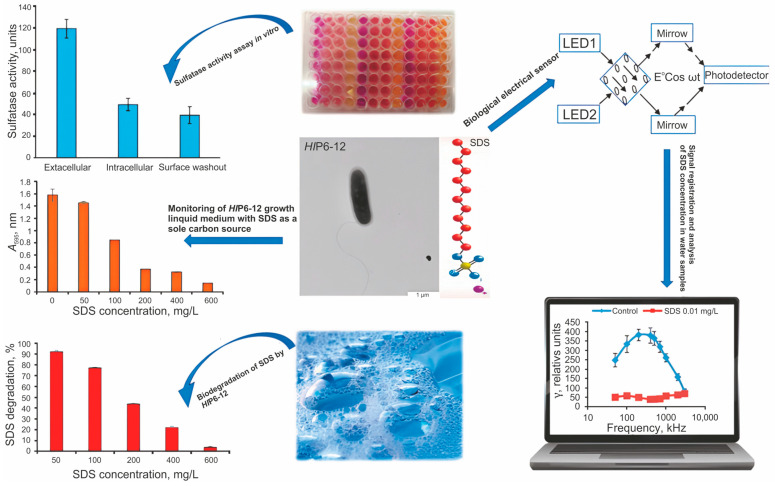
Microbial electric sensor for determination of sodium dodecyl sulfate based on gram-negative bacterium *Herbaspirillum lusitanum*, strain P6–12, as the sensing element. Reprinted with permission from ref. [139] Copyright © 2022, the authors, under exclusive license to Springer Nature B.V.

The publication [139] presents an approach to the detection of SDS, the predominant surfactant with significant environmental consequences. The study highlights the development of a biosensor using the gram–negative bacterium *Herbaspirillum lusitanum* strain P6-12, which can metabolize SDS as a single carbon source (Figure 12).

The authors focused on the following three main aspects: bacterial strain selection, sensor development, and experimental setup. The researchers identified *H. lusitanum* P6–12 by its ability to utilize SDS concentrations ranging from 0.01 to 400 mg/L. The sensor performance was evaluated by measuring the electrical polarizability of bacterial cell suspensions at frequencies from 50 to 3000 kHz. The achieved detection limit turned out to be surprisingly low and amounted to 0.01 mg/L, exceeding the regulatory standards for drinking water (0.5 mg/L) and industrial water (1.0 mg/L). As a result of their research, the research team concluded that the developed sensor was able to effectively detect low concentrations of SDS in aqueous solutions for 1–5 min, and the electrical polarizability of bacterial cells changed significantly in response to changes in SDS concentrations, indicating a strong correlation between the presence of SDS and the sensory response. The authors discuss the possibilities of using the biosensor not only for environmental monitoring but also for carrying out restoration work on SDS-contaminated soils. They highlight the advantages of using microbial biosensors, including their specificity, fast response time, and lower operating costs compared to traditional methods.

The disadvantage of many of these works is the lack of optimization of sensors for specific surfactants. Many sensors do not distinguish between individual surfactants or detect a mixture of organic substances, which makes it difficult to identify and quantify specific substances. Environmental conditions (temperature, pH, salinity, and the presence of other substances) significantly affect the accuracy and reproducibility of the results, so it is necessary to optimize sensors for real-time monitoring. These shortcomings emphasize the need for further research on the development of more effective and reliable methods for the determination of surfactants. In conclusion, it should be noted that the detection of surfactants in wastewater and their management using microbial sensors and biodegradation processes are crucial for maintaining the integrity of the environment. Advances in sensor technology and a deeper understanding of the interaction of microorganisms with surfactants will contribute to more effective wastewater treatment strategies, ultimately reducing the environmental risks associated with contamination with surfactants.

### 3.5. Phenols

Monitoring the content of phenol and phenolic compounds in water is important because of its widespread use in various industrial fields. Such compounds are used in the production of polymers, pesticides, medicines, various resins, and other organic compounds based on phenol, which leads to pollution of wastewater [146,147,148,149]. Due to its high toxicity, phenol is recognized by the World Health Organization as a group 2B carcinogen, which should not exceed a concentration of 1 µg/L in drinking water [150]. Worldwide, the maximum permissible concentrations (MPCs) range from 1 µg/L to 1000 µg/L, depending on the type of water source and the impact on the ecological system.

Various methods are used to determine phenol in water, such as the photometric method [151], the gas–liquid chromatography method [152], the high-performance liquid chromatography (HPLC) method, and the fluorometric method [153]. However, these methods have a number of disadvantages (extraction, which leads to increased analysis errors, secondary contamination with the solvents used, as well as long sample preparation and analysis times) and high requirements for the operator. The modern world requires the timely detection of toxicants before they enter the human environment directly. For this, phenol monitoring must be carried out locally and with high sensitivity. Modern biosensors, both enzymatic and microbial, meet these requirements [42,146,154,155].

When creating microbial biosensors that can detect phenols, there are problems with the low selectivity and sensitivity of the systems being developed. The selectivity of the systems is increased by means of adaptation of microorganisms to phenols, and the sensitivity is increased using carbon nanotubes [155,156]. The narrowing of substrate specificity is also achieved by isolating the membrane fraction from microorganisms [42]. However, one of the modern solutions to these problems is the use of genetic engineering to introduce plasmids and create mutant bacterial strains for the determination of phenols [157,158]. Next, we will discuss each of these approaches in detail.

**Figure 13:**
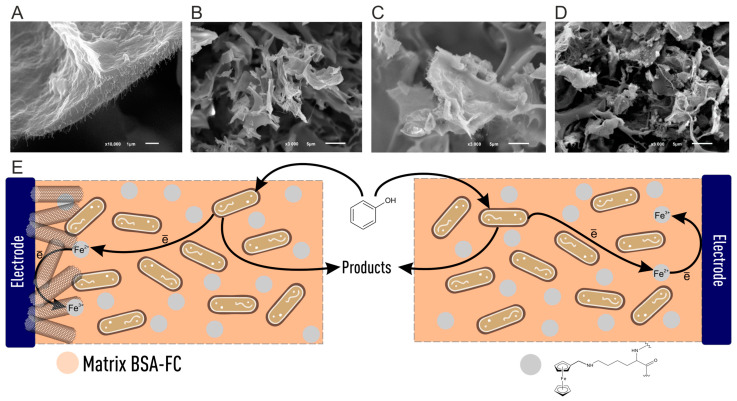
(**A**–**D**) SEM photographs of the BSA-FC/CNT matrix at various stages of modification; (**E**) the electronic transfer mechanism in the matrix. Reprinted from ref. [155] © 2022 by the authors.

The goal of all adaptation-based work is to increase the selectivity of microbial-based systems. Successful adaptation is achieved by using mineral media and gradually replacing natural carbon sources until phenol becomes the only source of carbon for microorganisms. This method is the least financially costly as there is no need to use genetic engineering methods. However, full adaptation (narrow specificity) cannot be achieved without the use of new materials for immobilization and carbon nanomaterials. Scientists have developed a fast, sensitive, and miniature microbial conductometric biosensor for the determination of phenol based on *Pseudomonas* sp. (GSN23) cells and modified gold microelectrodes with multi-walled carbon nanotubes (MWCNTs) [156]. The long-term stability of the developed system is only 5 days, and then a sharp decrease in sensitivity is observed. The paper presents substrate specificity only for phenolic components, and there are no other substrates that may be present in real wastewater. Therefore, the results may not be accurate enough to use such sensors in real samples. After adaptation, microorganisms still continue to respond to other substrates from the class of carbohydrates and alcohols, as shown in [155]. However, scientists achieve a narrowing of substrate specificity by immobilizing an adapted *Pseudomonas putida* culture into a BSA-FC/CNT composite polymer and suggest that phenol is oxidized by enzymes located directly on the outer membrane of cells. Covalently crosslinked ferrocene performs the function of electron transfer from the cell surface and, therefore, does not have time to achieve a response from other substrates (Figure 13).

However, the determination of phenol is also possible by microorganisms that are not adapted to phenol if isolates from soils or waters are used. For example, isolates of *Pseudomonas aeruginosa*, *Staphylococcus sciuri*, and *Bacillus amyloliquifaciens* were used in [159] to form biofilms and determine phenol and polyaromatic hydrocarbons. However, genetic analysis showed that the phenolic degrading oxygenase gene was found only in *P. aeruginosa* and *S. sciuri*. The limit of their detection is in the range of 0.5–1 µM. In a recent study [146], a copper electrode was used and three types of electrochemical signals were compared—EIS, square wave, and cyclic voltammetry. The aim of this work was to combine two methods of phenol destruction, the electrochemical method, which is often blocked by poisoning of the electrode surface due to the formation of reaction intermediates, and the biological method based on the biodegradation of phenol by bacteria. A new method of immobilization of Staphylococcus aureus bacteria on a copper electrode modified with a polycaprolactone film is presented. The linear range is from 0.01 to 0.05 M. However, in this work, it is again not possible to oxidize a range of other organic compounds that may contribute to the emerging biosensor response in real samples.

Microorganisms isolated from industrial wastewater and grown on a single carbon source, phenol, can also be used, as in [160]. An MFC based on a biofilm of microorganisms was used as a biosensor. The parameters of the microbial fuel cell were optimized using mathematical modeling: an improved ANN model was developed to predict phenol biodegradation. Here, a scaled conjugate gradient algorithm was used to predict and optimize the performance of MFC-based sensors by changing input parameters such as the concentration of the phenol substrate and the composition of synthetic wastewater. This approach is of practical importance, since when using MFCs, not only is the continuous monitoring of phenol in water achieved but also the generation of bioelectricity. However, the minimum concentration of phenol, which was determined in the work, was 100 mg/L, and the maximum achieved power was 395 ± 8 mV.

**Figure 14:**
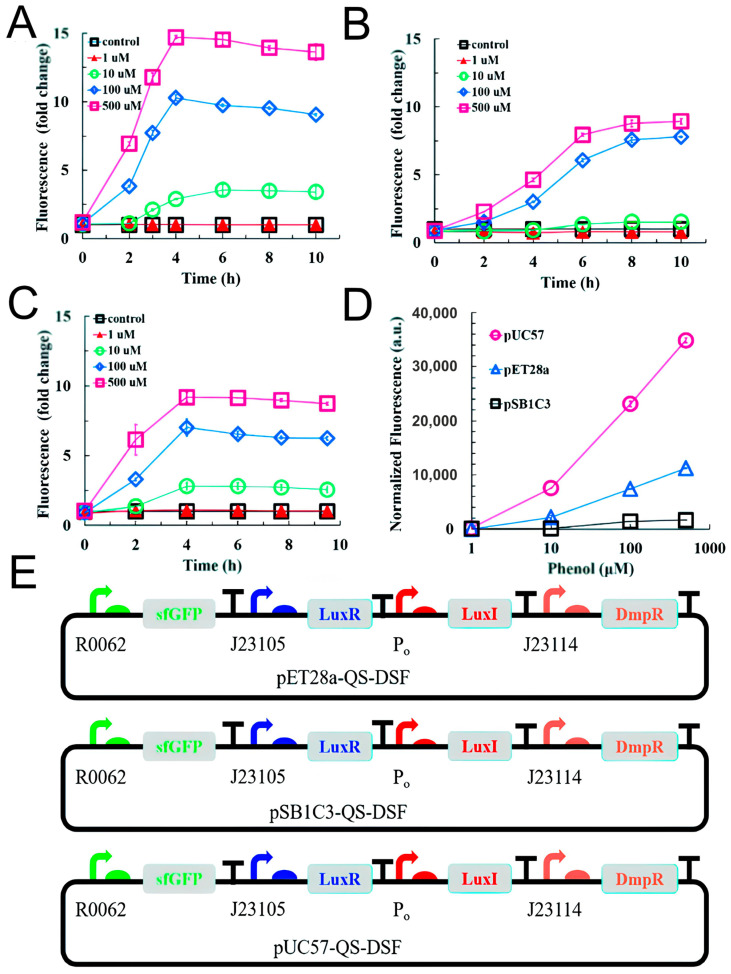
Determination of phenols in the range of 1, 10, 100, and 500 µm using microbial biosensors (**A**) pET28a-QS-DSF/*E. coli* DH5α; (**B**) pSB1C3-QS-DSF/*E. coli* DH5α; and (**C**) pUC57-QS-DSF/*E. coli* DH5α; (**D**) dynamic ranges of three biosensors in phenol of 1–1000 µM; (**E**) plasmids for phenol detection in this study: pET28a-QS-DSF, pSB1C3-QS-DSF, and pUC57-QS-DSF. Reprinted with permission from ref. [157] © 2022 Elsevier B.V. All rights reserved.

High success has been achieved in the works devoted to the genetic modifications of bacteria, such as increased sensitivity and the narrowing of substrate specificity. For example, in one of the studies, the microbial biosensor pUC57-QS-DSF-F42 L/*E. coli* DH5α was designed, which can recognize a wide range of phenolic compounds including phenol and p-nitrophenol (Figure 14). Due to the optimization of genetic chains, the detection limits of phenol and p-nitrophenol reached 0.1 and 1 µM, respectively [157].

The detection limit of phenol was reduced to 0.1 µM with a range of 0.1–500 µM. The ability to oxidize other phenols was noted, which falls in the range of o-cresol, o-nitrophenol, 1-naphthophenol, p-nitrophenol, m-cresol, resorcinol, hydroquinone, and 2-aminothiophenol.

**Figure 15:**
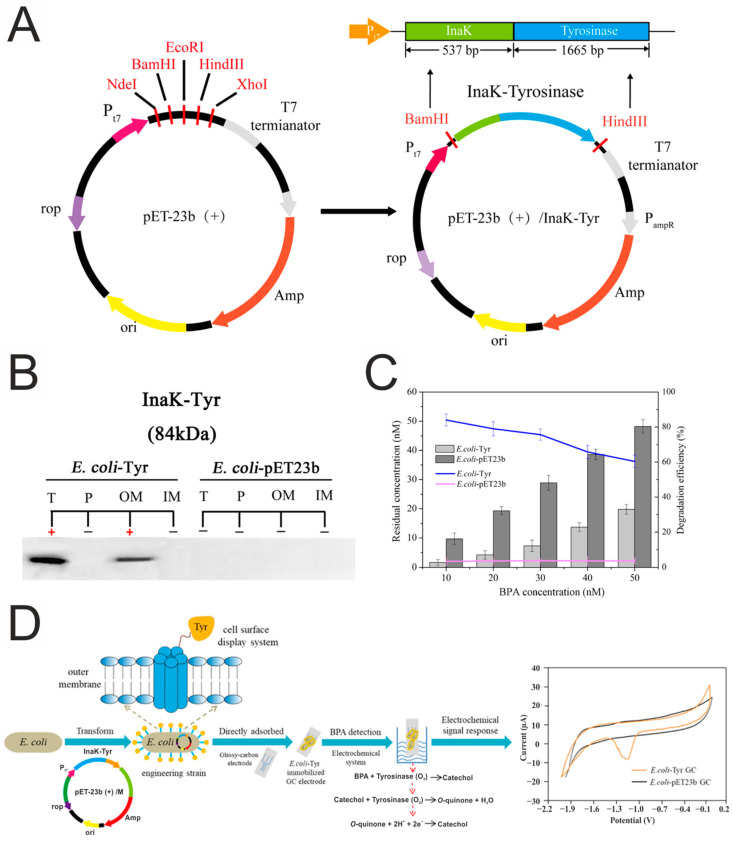
(**A**) Schematic illustration of the construction of the recombinant pET23b-Tyr plasmid and a gene map of the InaK-Tyr fusion gene; (**B**) analysis of the surface location of InaK-Tyr by Western blotting with various cell fractions; (**C**) reduction of BPA conversion using engineered *E. coli*-Tyr. Residual concentrations of BPA and the efficiency of decomposition at various concentrations; (**D**) the scheme of formation and operation of a biosensor for the determination of BPA. Reprinted with permission from ref. [161] © 2021 Elsevier B.V. All rights reserved.

In the study [161], an electrochemical biosensor was developed for the determination of bisphenol A (BPA) (Figure 15). The biosensor is based on the immobilization of tyrosinase on the surface of *Escherichia coli* cells, which are then adsorbed on a glass–carbon electrode. The biosensor demonstrated a linear relationship between BPA concentration and current strength in the range from 0.01 to 100 nM (correlation coefficient R^2^ = 0.9967). To verify the accuracy of the biosensor, BPA measurements were performed on real tea and juice samples. The results showed high accuracy, comparable to the results of the HPLC analysis.

An increase in the expression of the *carE* gene encoding catechol 2,3-dioxygenase was cloned and overexpressed in *E. coli* BL21, which led to the selective determination of catechol [43]. However, the authors of the article themselves note that for environmental monitoring in future work a microbial biosensor that could simultaneously detect several pollutants is urgently needed.

**Figure 16:**
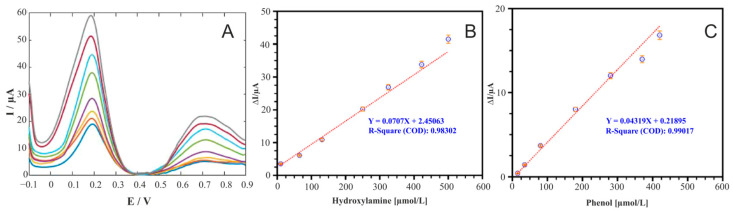
(**A**) Graphs of DHP/MgO NPS/CPE differential pulse voltammetry in PBS (0.1 M, pH = 7.0) containing various concentrations of hydroxylamine (10.0–500.0 µM) and phenol (150.0–420.0 µM); (**B**) graph of peak current depending on the volume of hydroxylamine (slope = 0.0782 µA); (**C**) graph of the peak current as a function of the volume of phenol (slope = 0.0393 µA/µM). Reprinted with permission from ref. [162] Copyright © 2024, King Fahd University of Petroleum and Minerals.

There are also works using nanoparticles to increase the sensitivity of the sensors being developed, for example, in [162]. It is important to note that this work considers the parallel determination of two analytes, phenol and hydroquinone. The linear range of phenol determination in the presence of hydroxylamine was 150.0–420.0 µM. An analysis of real drinking water samples was also carried out, to which different concentrations of phenol were added (Figure 16).

The microbial biosensors currently being developed for the determination of phenols are capable of simultaneously detecting several different analytes, have high sensitivity and a sufficient range to determine the MPC and lower. However, modern research still pays little attention to the analysis of real samples and the analysis of substrate specificity, especially of other organic molecules that may be present in the analyzed samples. Biofilm-based BESs [163] are beginning to be used both for phenol utilization and for detection. The use of carbon nanomaterials and nanoparticles successfully increases the sensitivity of microbial sensors but leads to an increase in their cost.

### 3.6. Pesticides

**Figure 17:**
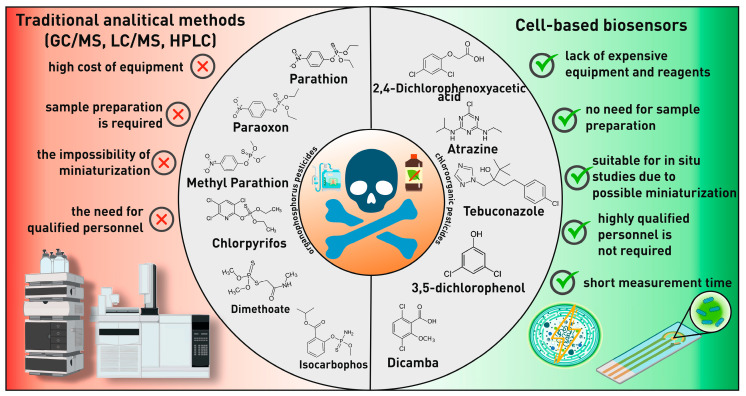
Advantages of microbial biosensors in comparison with traditional methods in the determination of pesticides.

Pesticides are chemicals necessary to protect crops from pests, diseases, and weeds. But their widespread use carries serious environmental and medical risks. Pesticides negatively affect biodiversity, pollute soil and water, and accumulate in the environment. For humans, they can cause diseases of the respiratory system, oncological diseases, and have a neurotoxic effect [164,165]. Strict control over the concentration of pesticides is necessary to minimize their harm. Currently, methods such as gas chromatography with mass spectrometry, liquid chromatography with mass spectrometry, and HPLC are used for this. However, these methods require expensive and bulky equipment, which makes their application difficult [166]. In search of a solution, scientists are actively developing new technologies, for example, sensors based on microbial biosensors. These devices are more compact, faster to use, and affordable, which makes them an ideal solution for field research (Figure 17) [167,168,169,170,171,172,173].

One of the extensive groups of pesticides are organophosphate pesticides (OPs). This section will review the current state of technologies for detecting organophosphate pesticides using biosensors.

**Figure 18:**
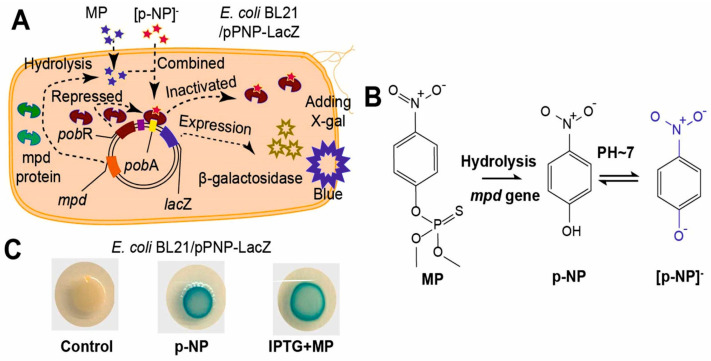
Operation of a microbial paper biosensor for reaction to p-nitrophenol and methylparathion. (**A**) The genetic organization and mechanism of methylparathion; (**B**) the chemical structure of target effector molecules ([p-NP]^−^) for the *pob*R transcription factor, binding domains [p-NP]^−^ to the *pob*R transcription factor; (**C**) the color reaction of bacteria (*E. coli* BL21/pPNP-lacZ) induced by 10 mg/L of p-nitrophenol and 10 mg/L of p-nitrophenol with 1 mg/L of IPTG. Reprinted with permission from ref. [174] © 2023 Elsevier B.V. All rights reserved.

The use of genetic engineering can be noted. For example, Zhao Ma and colleagues have developed a biosensor for the determination of methyl parathion (MP) based on a modified strain of *E. coli* immobilized with alginate on a paper strip. The principle of action is based on the hydrolysis of MP to para-nitrophenol (p-NP), regulated by the modified mpd gene (Figure 18A).

The addition of isopropyl-β-D-thiogalactopyranoside (IPTG) causes active gene expression, resulting in the production of hydrolases interacting with MP. The resulting p-NP then interacts with the *pob*R transcription factor in a specific way, which eventually leads to the production of β-galactosidase (Figure 18B). The enzyme β-galactosidase can destroy 5-bromo-4-chloro-3-indolyl-β-D-glucopyranoside (X-gal), resulting in a blue spot (5.5′-dibromo-4,4′-dichloro-indigo, Figure 18C). The color intensity was evaluated using the ColorMeter app and was then used as an analytical signal depending on the MP concentration. The developed biosensors with a paper strip are capable of quantifying concentrations of 10–10,000 µg/kg for p-NP and 25–7500 µg/kg for MP with a detection limit of 5.41 µg/kg for p-NP and 9.57 µg/kg for MP in soil [174].

A microbial biosensor based on modified *E. coli* and quorum sensing (QS) was also developed [157]. Quorum sensing is a special genetic mechanism used by many bacteria to communicate and coordinate population behavior by producing and detecting secreted molecules in the environment. This biosensor allows not only to detect MP but also to carry out its complete biodegradation. For this purpose, the researchers implemented the QS module between the phenol detection module (DmpR) and the reporter module (sfGFP or OPH) of the microbial biosensor. When MP is added, its spontaneously hydrolyzed p-NP product can bind to the developed DmpR, inducing LuxI protein expression, causing the synthesis of N-acylhomoserinlactone (AHL). AHL then binds to the LuxR protein and activates the expression of modified OPH, which destroys MP and generates more p-NP, forming a positive feedback loop. This process can be repeated until the entire MP is destroyed and the cell can grow continuously after MP biodegradation. After replacing the reporter protein with the MP-destroying enzyme PoOPHM2, the biosensor pUC57-OPH-QS-DSF-F42 L/*E coli* DH5a was able to detect the presence of MP and actively express enzymes to destroy MP when the MP concentration reaches a threshold value. Five consecutive decomposition experiments have shown that 10 µM and 100 µM of MP can be completely decomposed within 8 h, indicating their potential for continuous purification of contaminated water.

One of the developing directions is the use of the enzyme acetylcholinesterase (AChE) for the determination of organophosphate pesticides [175,176,177,178,179]. However, obtaining the enzyme is laborious, and acetylcholinesterase has low sensitivity to pesticides. In this regard, researchers are looking for ways to increase the sensitivity and expression of AChE, as well as its stability. In [180], microbial surface display is used for this purpose as a method of enzyme immobilization on the cell surface. This method involves the genetic engineering of exogenous peptides in the form of a fusion protein that is displayed on the cell surface and retains its relatively independent spatial structure and biological activity. In this work, the AChE gene isolated a carp was mapped on the surface of *S. cerevisiae* using α-agglutinin as a motif for fixation. The choice of carp as a source of the enzyme is due to the fact that this fish is a biomarker of the aquatic environment contaminated with OP, therefore the enzyme has a high sensitivity. The determination of OP—parathion and paraoxon—was carried out by evaluating the rate of AChE inhibition during the incubation of a recombinant strain with different concentrations of pesticides. The detection limits were 0.136 ng/mL of paraoxon and 3.72 ng/mL of parathion, respectively.

To determine paraoxon in [181], a biosensor based on *E. coli* expressing fusion proteins consisting of organophosphorus hydrolase (OPH) and the pH-sensitive green fluorescent protein pHluorin was used. The authors examined several combinations of proteins, including those linked by the linker (L), and the most effective was the fusion protein pHluorin-L-OPH with a detection limit of 0.5 µg/mL.

A biosensor based on the use of a formylglycine-generating enzyme (FGE) and a sulfatase system consisting of a chpA promoter fused with FGE sulfatase reporter genes (pChpAp–atsBA) has been proposed for the detection of chlorpyrifos. Once *E. coli* is exposed to CPF, activated ChpR activates FGE (atsB) expression and sulfatase (atsA) genes through the chpA promoter. FGE then changes the single serine sulfatase residue at position 72 to formylglycine. The activated sulfatase then cleaves the sulfate group from 4-methyllumbeliferon sulfate (4-MUS) to produce the fluorescent product 4-methyllumbeliferon (4-MU). By modifying this system, the authors managed to achieve a detection limit of 5 nM [182].

Organochlorine pesticides are another large group. Despite the fact that many of them are prohibited for use, some of the residual organochlorine pesticides and their transformation products are still found in environmental objects [165].

To detect atrazine, a biosensor based on an artificial biofilm of photosynthetic microorganisms, *Anabaena veriabilis*, was proposed in [183], which measures the photocurrent during the inhibition of photosynthesis by pesticides. Since pesticides block the plastoquinone complex in photosystem II, an artificial mediator, para-benzoquinone, was used to control the photocurrent. The detection limit for atrazine was 0.07 µM.

**Figure 19:**
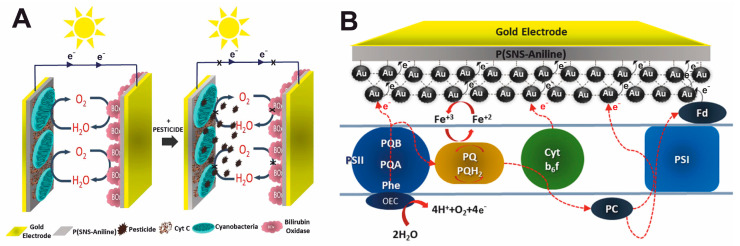
(**A**) Schematic representation of a BPV-based biosensor for detecting pesticides in water; (**B**) schematic representation of the electron transfer mechanism from the reaction center to the electrode. Reprinted with permission from ref. [184] Copyright © 2024 the authors. Published by the American Chemical Society.

In another work, a biological photovoltaic cell (BPV) based on cyanobacteria *Leptolyngbia* sp. was constructed, which the authors used as a biosensor for the determination of atrazine (Figure 19) [184]. Its action is also based on the inhibition of the photocurrent by pesticides.

The biocathode in this system was modified by crosslinking the enzyme bilirubin oxidase (BOx) with poly-4-(2,5-di(thiophene-2-yl)-1H-pyrrole-1-yl)-benzamine (P(SNS-aniline)). *Leptolyngbia* sp. was applied to a bioanode, which is a gold electrode modified with gold nanoparticles (AuNPs) and P(SNS-aniline). (Figure 19A). P(SNS-aniline) and AuNPs serve as mediators that establish close interactions with cyanobacteria and molecular contact with the outer membrane. Their redox centers also facilitate the transfer of electrons to the electrode. The possibility of electron capture occurs when P(SNSAniline)/AuNPs pass through the pores of the outer membrane, potentially reacting with charged molecules through proteins located in the cytoplasmic membrane (Figure 19B). The BOx enzyme, crosslinked with a biocathode, promotes the bioelectrochemical conversion of oxygen into water. The resulting system has a detection limit of atrazine equal to 0.014 µM.

An optical biosensor based on whole cells of the green photosynthetic algae *Chlamydomonas reinhardtii* detects nanocapsulated atrazine by changes in the fluorescence emission of algae chlorophyll [44]. The algae were immobilized on a paper substrate using an agar hydrogel. The detection limit of nanocapsulated atrazine was 4 pM.

A microbial fuel cell proposed by Jon Chouler and Mirella Di Lorenzo in [185] can also be used to determine atrazine. The device was a single-chamber membrane-free MFC, where the MFC was coated with a biofilm of a mixed culture of electrochemical active microorganisms from anaerobic sludge. The developed MFC biosensor makes it possible to detect atrazine with a detection limit of 0.05 ppm.

The previously mentioned sulfatase system was used by Benjarat Ritcharoon and colleagues to develop a biosensor for 2,4-dichlorophenoxyacetic acid (2,4-D) [186]. *Agrobacterium tumefaciens* with a *cadA* promoter and a *cadR* regulator from the *Bradyrhizobium* sp. HW13 strain with an (FGE) sulfatase as a reporter gene were taken as the host strain. The sensitivity of the biosensor was improved by using the directed evolution of *cadR* by random mutagenesis. The detection limit for 2,4-D was 1.56 µM.

Another common fungicide is tebuconazole (TEB). To determine it, in [187] they increased the expression of the reporter gene, nanoluciferase, by *ERG* promoters in *S. cerevisiae* yeast. *ERG* promoters encode ergosterol biosynthesis, and when exposed to TEB, they are expressed and the bioluminescence signal is increased. The developed biosensor is specific to TEB and has a detection limit of 5 µg/L. To detect dicamba herbicide, Eric VanArsdale and colleagues genetically modified *E. coli* [188]. They enhanced the expression of the *lacZ* gene which is mediated by the soxRS regulon encoding β-galactosidase. Diquat can also activate soxRS-mediated β-galactosidase expression. This enzyme can be used in combination with ortho-nitrophenyl-β-galactoside (OPNG) or 4-aminophenyl β-D-galactopyranoside (PAPG) to produce an optical or electrical analytical signal, respectively. In the case of both optical and electrochemical detection, the linear range was 0–4.5 mM.

Reactions of microorganisms to the effects of toxicants can be assessed using Raman spectroscopy [189,190,191,192,193]. For example, an automated spectroscopic system was proposed in [194], where the principle is based on monitoring the effects of a toxicant on the Raman spectra of cellular components. For *E. coli* MG1655 exposed to various concentrations of 3,5-dichlorophenol, characteristic changes are observed for bands associated with phenylalanine (1000 cm^−1^) and phosphate groups of DNA–PO_2_ (1100 cm^−1^). The intensity of these bands decreases with increasing concentration of the toxicant. This method allows the determination of 3,5-dichlorophenol in the range of 2.5–250 mg/L.

The development of microbial biosensors for the determination of pesticides is dynamically developing, based on the achievements of genetic engineering, which not only enhances the expression of the necessary genes but also use quorum sensing to increase the sensitivity of biosensors. The use of microbial surface display technology for enzyme immobilization makes it possible to create more stable and sensitive biosensors, overcoming the disadvantages of traditional enzyme systems. Photovoltaic technologies based on cyanobacteria represent a promising area that allows for not only detecting pesticides but also producing energy. In addition to monitoring toxicants, Raman spectroscopy allows for a deep study of their effect on microorganisms, which contributes to the development of more advanced biosensor systems.

### 3.7. Inorganic Pollutants

Microbial electrochemical biosensors are an effective analytical tool for detecting a wide range of inorganic substances that pollute the environment, including not only heavy metals, but also elements such as arsenic and antimony. Their principle of operation is based on the use of genetically modified microorganisms that express reporter genes that are sensitive to concentrations of target ions. The general detection mechanism includes several stages: preparation of cells, their exposure to test samples potentially containing target ions, and the registration of a measurable signal, the intensity of which correlates with the concentration of ions present. Thus, the biosensor provides the ability to quantify inorganic pollutants.

In particular, biosensors based on *E. coli* have been developed for the detection of arsenic and antimony, characterized by a minimum detection limit of 0.1 µM which is significantly lower than the permissible level for drinking water set by the World Health Organization (0.13 µM) [195]. This highlights their potential for use in monitoring the quality of water resources. Another example is a biosensor based on *Bacillus subtilis* spores using a reporter gene for a green fluorescent protein, which is activated in the presence of inorganic forms of arsenic (As^3+^ and As^5+^). This system is capable of detecting arsenic in the concentration range from 0.1 to 1000 µM within 4 h after spore germination, demonstrating a wide dynamic range and a response rate sufficient for practical use [196]. Studies also show the promise of using *Tetrahymena thermophila* as a biosensor for detecting heavy metals, including arsenic and Sb^3+^. The use of metallothionein promoters associated with luciferase genes provides these biosensors with the possibility of highly sensitive detection of bioavailable metals for about 2 h [197]. Thus, microbial electrochemical biosensors represent a versatile and promising platform for the analysis of a wide range of inorganic pollutants.

Microbial electrochemical biosensors are increasingly being used to detect key nutrients and pollutants in environmental samples, including nitrates (NO_3_^−^), phosphates (PO_4_^3−^), and sulfates (SO_4_^2−^). Studies demonstrate that these biosensors can effectively monitor nitrate and phosphate levels in various samples, providing real-time data necessary for water quality management [198]. In particular, a biosensor based on *E. coli* has been engineered to express a reporter gene sensitive to nitrate levels. This biosensor demonstrated a nitrate detection limit of 1 µM [199].

Biosensors based on microbial strains capable of metabolizing phosphate compounds have been developed to detect phosphates, which allows quantitative measurements of phosphate levels in the aquatic environment [200]. Studies show that *Bacillus subtilis* strains can be genetically modified to respond to phosphate levels by expressing a reporter gene associated with phosphate metabolism pathways, while achieving high sensitivity and specificity [201]. Microbial biosensors based on sulfate-reducing bacteria have been developed to detect sulfates in environmental samples. These biosensors use the metabolic activity of these bacteria, which convert sulfates into sulfides, to generate measurable electrochemical signals. One study reported a biosensor capable of detecting sulfate concentrations up to 10 µM using voltammetric methods, demonstrating high sensitivity and fast response time.

Despite the limited number of studies devoted to the detection of halides using biosensor technologies, there are a number of significant achievements in this field. Recent work has focused on the development of biosensors capable of simultaneously detecting multiple pollutants, including heavy metals and potential halides. Such multiplex systems have increased specificity and sensitivity, which makes it possible to monitor the environment in real time [196,199]. The integration of synthetic biology methods into the design of microbial biosensor systems has led to the creation of biosensors with improved detection capabilities for various pollutants, including inorganic compounds. This approach can be adapted to detect halides using genetically modified microorganisms that specifically react to these compounds [199,200].

Microbial electrochemical biosensors are particularly promising for monitoring the environment with regard to oxidizing agents such as perchlorate, which is a common contaminant of water sources. The use of genetically modified bacteria allows for the selective detection of specific oxidizing agents. For example, some strains can be modified to produce a measurable signal in response to exposure to perchlorate, thereby providing information about contamination levels [99,202]. Research is also focused on the development of biosensors that specifically respond to perchlorate ions. These developments often include modification of bacterial strains to generate bioluminescent or electrochemical signals when exposed to perchlorate, thereby providing a fast and effective detection method in environmental samples [202,203,204].

### 3.8. Viral and Microbiological Contamination

Indoor environments may contain a variety of airborne pathogens such as bacteria, fungi, yeast, mold, and viruses. These microorganisms can spread from aerosols released by ventilation systems, heaters, air conditioners, or humidifiers. They are constantly present in the environment, and their particles, regularly spreading, enter the indoor air. Various airborne pathogens can have a negative impact on human, animal, and plant health. Infectious diseases caused by these microorganisms pose a significant threat to health, reduce labor productivity, and lead to significant economic losses in various sectors [205].

The accurate and sensitive detection of pathogenic microorganisms remains a difficult task and is an important scientific problem [206]. Traditional methods of pathogen diagnosis based on the cultivation of microorganisms on agar media often take a long time, which reduces their effectiveness in clinical practice. Bacteria remain the most common pathogens detected by electrochemical biosensors, while the detection of viruses and protozoa has been the subject of active research in recent years. Typically, pathogens can be identified based on the detection of specific antibodies produced by the body in response to infection. In such analyses, antibodies act as an element of biological recognition and as an analytical target. The analytical mechanism used in biosensors to detect pathogens is based on the recognition of a specific biomarker specific to each pathogen using an immobilized sensitive material called a “bioreceptor”. A bioreceptor can be a monoclonal antibody, RNA, DNA, glycan, lectin, enzyme, tissue, or an entire cell. The bioreceptor must have biochemical properties that ensure high sensitivity and selectivity when detecting a biomarker, thereby eliminating the influence of other microorganisms in the analyzed sample. The specific biochemical interaction between the biomarker and the bioreceptor is transformed into a measurable signal recorded by the detector [207].

A variety of biological objects are used as bioreceptors in biosensor systems. For example, peptides forming self-organizing monolayers on the surface of a gold electrode are used to detect human noroviruses that cause gastrointestinal infections in the form of acute gastroenteritis [206]. These systems demonstrate high selectivity when binding to norovirus, which is manifested in significant changes in the electrochemical signal.

**Figure 20:**
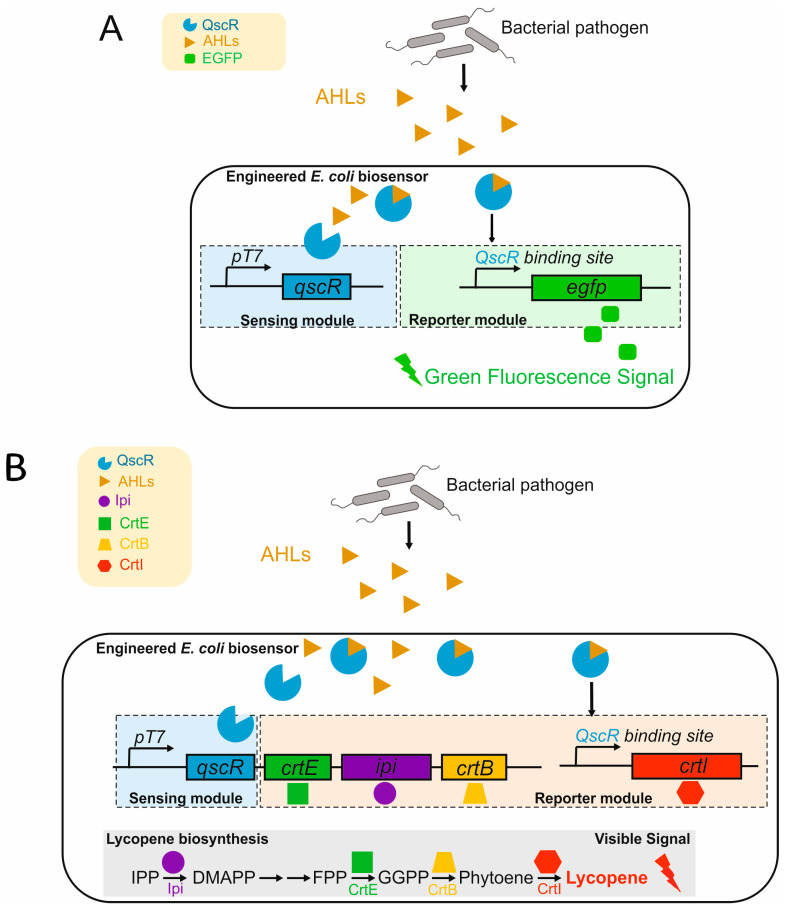
Schematic of engineered microbial biosensor containing the QscR sensing module coupled with an enhanced green fluorescence protein (*EGFP*) reporting module (**A**) or a lycopene reporting module (**B**). Abbreviations used are as follows: *QscR*, a transcriptional activator protein from *P. aeruginosa*; AHLs, N-acylhomoserine lactone; *CrtE*, geranylgeranyl pyrophosphate synthase; *CrtB*, phytoene synthase; *CrtI*, lycopene synthase; *Ipi*, isopentenyl pyrophosphate isomerase; IPP, isopentenyl pyrophosphate; DMAPP, dimethylallyl pyrophosphate, FPP, farnesyl diphosphate; GGPP, geranylgeranyl pyrophosphate. Reprinted with permission from [208]. Copyright © 2021 the authors. Published by the American Chemical Society.

Microbial biosensors play an important role in detecting waterborne pathogens such as *Escherichia coli* and *Pseudomonas aeruginosa*. In particular, research has developed a microbial biosensor capable of detecting *Pseudomonas aeruginosa* and *Burkholderia pseudomallei* based on quorum sensing signaling molecules, which demonstrates high sensitivity and specificity to these pathogens in water samples [208]. An *E. coli*-based biosensor detects bacteria by AHL signals. The sensor module, controlled by the pT7 promoter, expresses *QscR*. When linking to AHL, QscR activates the reporter module. In variant (A), activation leads to *egfp* expression and fluorescence. In variant (B), egfp is replaced by the *crtE*, *ipi*, *crtB*, and *crtI* genes encoding lycopene synthesis. Activation leads to the production of lycopene and a color change to red, indicating the presence of bacteria (Figure 20).

Microbial biosensors are also promising for detecting viruses such as enteroviruses and noroviruses in water sources. Studies have shown the possibility of using electrochemical sensors to detect viral RNA, which may indicate the presence of pathogenic viruses in wastewater and drinking water supply. The ability to detect viruses quickly is crucial for public health. Microbial biosensors can be designed to respond specifically to viral pathogens, providing means for real-time monitoring in environmental conditions [198,208,209,210].

Microbial biosensors are also used to detect mycotoxins in environmental samples. Mycotoxins, which are toxic metabolites of fungi, pose a significant health risk and can contaminate food and feed. Biosensors of this type allow the assessment of mycotoxin contamination in environmental samples such as soil and water, providing a means for the rapid screening of contaminated sites, which is an important aspect for ensuring food safety and public health [198,199,211,212].

A separate area of research is related to the use of monoclonal and polyclonal antibodies in biosensor systems. In one study, a biosensor was developed to detect *Cryptosporidium*, which is the causative agent of cryptosporidiosis. In this case, specific monoclonal antibodies were covalently immobilized on the surface of gold electrodes using bovine serum albumin to quantify *Cryptosporidium* oocysts in water samples [213]. In another study, specific polyclonal antibodies were used to detect *E. coli*. During the formation of the biosensor, silicon nanoparticles were deposited on the gold electrode, and then on the antibodies themselves. This modification made it possible to increase the detection sensitivity of *E. coli* compared to earlier developments [214].

In addition, biosensors for detecting various pathogens, including those using the molecular imprinting method, are presented in research papers. For example, biosensor systems have been developed for the recognition of *E. coli* using ultrathin sol–gel film coatings [215], and for the detection of *S. aureus* using a silver-based matrix decorated with manganese dioxide and a thin-film electrode made of tin oxide doped with fluorine [216].

## 4. Commercial Bioanalytical Systems Based on Whole Cells of Microorganisms

**Figure 21:**
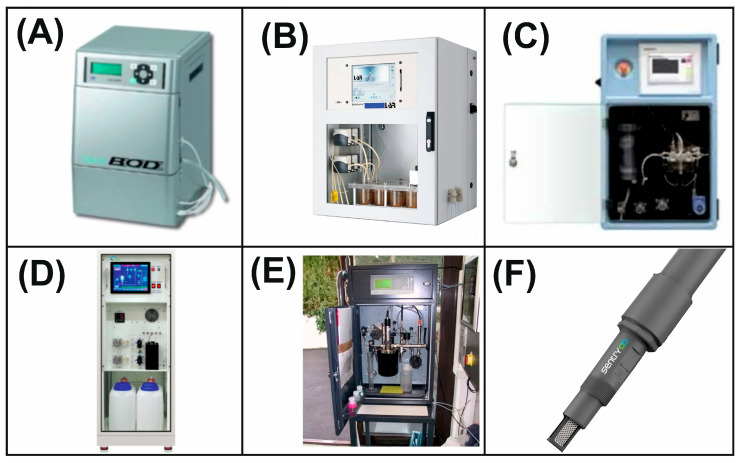
Commercial biosensors for determination of BOD: (**A**) Central Kagaku Biosensor Analyzer, BOD α1000; (**B**) BioMonitor™; (**C**) Ra-BOD; (**D**) HABS-2000; (**E**) C-Sys Biox 1010; (**F**) SENTRY™ Standard.

Despite their promising characteristics and the availability of a large number of exciting prototypes, microbial biosensors are struggling to make the transition from laboratories to commercialization. Their main application has been for water quality assessment and monitoring of water treatment quality from various pollutants. The most commercialized among such biosensors are those for the determination of BOD. Generally, such devices are based on a Clark-type oxygen electrode and the reaction of microorganisms is measured in a reactor. The organic content of the wastewater affects the metabolic activity of the microbial cells contained in the receptor, resulting in an increase in respiration or electrons, which in turn affects the biosensor signal. This allows real-time determination of the water treatment efficiency and its individual characteristics. The first such analyzer was produced by Nisshin Electric Co. Ltd. (Kyoto, Japan) in 1983, and then in 1990 the biosensor method for BOD determination was included in the Japanese Industrial Standard (JIS, JIS K3602) [217]. At present, commercial BOD biosensors are produced by many European firms: (Dr. Lange GmbH (Hamburg, Germany), Aucoteam GmbH (Berlin, Germany), and Prufgeratewerk Medingen GmbH (Ottendorf-Ocrilla, Germany), as well as Canadian (Sentry, PE, Canada) and Japanese manufacturers (Central Kagaku Corp., Tokyo, Japan). Manufacturers are focused on extending the range of measurable concentrations, reducing analysis time and increasing bioreceptor resistance to toxicants. For example, the MB-BOD device (patented and supported by several Spanish organizations) can sustain over 210,000 h of uninterrupted operation and determine effluent BOD [218] in about 1 h. The same company produces the POLYTOX-RES device, also based on various microorganisms, which allows for the observation of any type of water pollution, both organic and inorganic, in the range of 10–1000 mg/L of pollutant [219]. The Central Kagaku Biosensor Analyzer, BOD α1000 [220] is based on the principle of microbial respiration estimation, and its concentration range is 2~50 mg/L; BioMonitor™ [221], 1–200,000 mg/L BOD; Ra-BOD [222]; HABS-2000 [223], 0–200 mg/L; C-Sys Biox 1010 [224] 20 to 1500 mg/L; SENTRY™ Standard [225] 0.1 to 25,000 mg/L (Figure 21).

**Figure 22:**
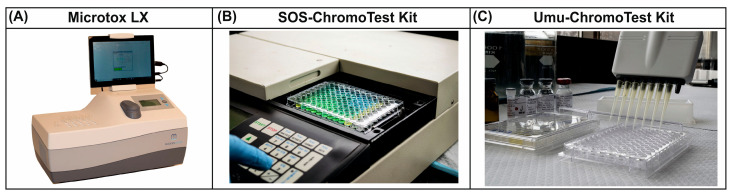
Commercial biosensors for determination of toxicity: (**A**) Microtox LX; (**B**) SOS-ChromoTest Kit; (**C**) Umu-ChromoTest Kit.

The ability to determine BOD is based on the metabolism of microorganisms and their broad substrate specificity and many pollutants lead to a decrease in the activity of luminescent microorganisms, which can be detected using optical devices. For example, many bacteria contain luminescence genes, and luminescence decreases in the presence of toxic substances in proportion to their content in the sample. The incorporation of luminescence genes into other species and genera of microorganisms from the original microorganisms (usually marine species like *Vibrio fischeri*) leads to an increase in the number of living objects used for toxicity assessment, as well as an expansion of possible analytes. This principle is the basis for the commercial biosensor ITOXcontrol [226] for measuring total water toxicity, BIOCARTE for the presence of xylene in samples [227], and Microtox LX [228] which is sensitive to more than 2700 simple and complex chemicals and allows for the protection of drinking water supplies from accidental or deliberate contamination (Figure 22A).

Toxicity can be tested not only based on the luminescence of microorganisms, but also on changes in their electrochemical properties. For example, electron transfer via reduction of a potassium ferricyanide mediator due to metabolism by indigenous bacteria in response to the introduction of toxicants became the basis of commercial biosensors SciTOX-ALPHA and SciTOX-UniTOX for the determination of 3,5-dichlorophenol (DCP), acetone, and some heavy metals. The CellSense biosensor system [229] provides almost continuous monitoring (measurement at 4 s intervals) of microbial activity after toxic shock, after addition of four heavy metal ions Hg^2+^, Cu^2+^, Zn^2+^, and Ni^2+^, and while the behavior of the biosensor electrochemical signal (amplitude, velocity, etc.) is different for each ion, which allows for the diagnosis of individual ions in the measuring medium.

It is well established that toxic compounds have the capacity to induce various mutations and are thus considered genotoxic. These mutations sometimes lead to the inhibition of certain metabolic processes, which can be monitored by biosensors. Commercial biosensors are being developed to determine the genotoxicity of various compounds. For instance, the SOS-ChromoTest^TM^ [230] (Figure 22B) employs a colorimetric assay in *Escherichia coli* to measure the expression of genes induced by genotoxic agents; this is achieved through a fusion with the β-galactosidase structural gene. Another approach, Vitotox [231], utilizes bacteria (TA 104-recN2-4 or Genox strain) containing the *Vibrio fischeri lux* operon regulated by the mutated *recN* promoter, which is under the control of the bacterial SOS system. When these cells are incubated with a genotoxic substance, the *recN* promoter is derepressed, leading to *lux* operon expression and light production proportional to the genotoxicity. False positives can occur if compounds directly affect light production (e.g., aldehydes) or enhance bacterial metabolism. Consequently, a control strain (pr1 or Cytox strain) with a constitutively expressed *lux* operon (pr1-lux fusion, yielding continuous light) is used. An increase in light from this Cytox strain indicates the compound affects the *lux* system via non-DNA damaging pathways, while a decrease suggests cytotoxicity. Furthermore, the Umu-ChromoTest kit (umu-c test) [232] relies on a specially engineered *Salmonella typhimurium* strain to measure the cellular response to genetic damage (Figure 22C).

Attempts are being made to develop commercial biosensors to detect potentially harmful compounds such as hormones. For example, the EstraMonitor for monitoring estrogens in wastewater and other samples, with a measurement range of 5 to 100 ng/L and a detection limit of 5.3 ng/L, is based on recombinant cells of the yeast *Arxula adeninivorans* as a biocomponent.

However, with all the variety of solutions available on the market, it is worth noting that the share of devices using live microorganisms as bioreceptors is still quite small. Moreover, some devices that used this approach could not survive the competition and have already disappeared from the market. There have been attempts to understand why microbial biosensors have had such a hard time becoming commercialized [233]. We believe there are three main limitations to the adoption of such devices.

Firstly, the inherent properties of microorganisms themselves impose certain limitations on device operation, such as pH, temperature, salinity, and other parameters. This is especially evident in the detection of pesticides and heavy metals, which are often toxic to microorganisms, especially at high concentrations. This can lead to degradation of the biological detection element, limitation of the dynamic range of the sensor, or saturation/decreased signal at high analyte levels. The stability of characteristics during operation also remains problematic, and there is a possibility of contamination by other microorganisms. Microorganisms have broad substrate specificity and another challenge in the detection of pesticides and heavy metals is that microbial biosensors can detect an entire class of pesticides (e.g., organophosphorus compounds) but cannot readily differentiate individual compounds within that class. Similarly, distinguishing between different heavy metals (e.g., Cd vs. Pb vs. Hg) based on general toxicity is difficult without highly specific engineered pathways. In such cases, it is necessary to involve specialists from other fields to build complex mathematical models that allow for the isolation of a useful signal for a specific object from the general response of the biosensor, or to involve AI.

Another point is that a significant proportion of laboratory models are based on genetically modified microorganisms. Although the genetically modified microorganisms have the advantage of providing specificity and selectivity to biosensors, they raise concerns about the feasibility of large-scale production of the devices due to challenges such as the instability of the genetic circuits, cell viability, and immobilization. In addition, there are concerns about environmental safety and the possibility of genetic contamination of non-targeted microorganisms.

Finally, there may be legislation, regulations, and existing specific standards for environmental monitoring that vary from country to country, and which often do not yet include microbial biosensors.

Researchers wishing to make a successful transition with their laboratory prototypes to the global market need to remember that biosensors must be designed first and foremost to be commercially viable. The development of cost-effective electrode materials and nanomaterials, the optimization of system design, and a reduction in power consumption are necessary to make microbial biosensors economically viable compared to conventional technologies.

## 5. Conclusions and Outlook

Of great importance for fundamental research and practical application of the created analytical devices is the verification of their operation at facilities designed for this purpose. The development of bioanalytical systems using whole microbial cells has attracted considerable attention due to the inherent advantages these systems offer in terms of sensitivity, specificity, and the ability to operate in challenging environments. These systems use the metabolic processes of microorganisms to convert chemical energy into electrical energy, which can be used for a variety of applications, including wastewater treatment and environmental remediation. The integration of exoelectrogens, such as *Pseudomonas* species, significantly increases the efficiency of these systems by facilitating electron transfer during microbial metabolism. In addition, the use of microfluidic technologies allows for the precise control of environmental conditions, ensuring simultaneous cultivation and analysis of microbial cells. The use of Raman spectroscopy combined with stable isotope sensing to identify metabolic states and interactions within microbial communities allows us to understand the role and contribution of each organism in complex ecosystems.

When it comes to biosensors that have found wide practical application, these are primarily biosensors for the determination of BOD. These biosensors are available in the commercial sector and have been incorporated into the regulatory frameworks of certain countries as they make it possible to significantly speed up the analysis of natural waters, control the operation of treatment plants, and monitor the quality of discharged water in accordance with standards. A notable limitation of such devices is the measurement of samples with high salt content, such as seawater. Research and development of cell immobilization methods for effective long-term stability of the developed devices, especially microbial fuel cells, is also ongoing.

An interesting approach that the authors draw attention to is called the application of quality by design principles in the development of bioanalytical methods; it is also gaining popularity, allowing a systematic approach to optimization and the validation of methods. By applying the principles of QbD, researchers can ensure that bioanalytical methods are not only effective, but also comply with regulatory standards, which facilitate their application in clinical and environmental settings. Two technologies that can contribute to the development of microbial biosensors should be noted: the introduction of artificial intelligence and Internet of Things technologies.

The use of genetically engineered microorganisms in biosensors is rapidly developing due to their increased sensitivity and specificity for detecting specific pollutants. For example, researchers have successfully developed microbial biosensors by combining luminescent genes such as *lux*, *gfp*, or *lacZ* with gene promoters that respond to the presence of heavy metals in water. The development of mediator microbial biosensors has also been studied as a means of increasing the sensitivity and specificity of toxicity detection in surface waters.

The versatility of microbial biosensors is confirmed by their use to detect specific environmental pollutants such as mercury and cadmium. These biosensors emit quantifiable signals in response to intracellular concentrations of these metals, providing valuable information about their bioavailability and potential toxicity.

The use of microbial biosensors goes beyond environmental monitoring; they are also used in food safety and quality assessment. For example, biosensors have been developed to detect pesticide residues and other pollutants in food, ensure compliance with safety regulations, and protect public health. Thus, microbial biosensors represent an advanced approach to toxicity detection that uses the natural capabilities of microorganisms to effectively monitor environmental pollutants.

Microbial biosensors are also promising for detecting viruses such as enteroviruses and noroviruses in water sources. Studies have shown the possibility of using electrochemical sensors to detect viral RNA, which may indicate the presence of pathogenic viruses in wastewater and drinking water supply. Microbial biosensors are also used to detect mycotoxins in environmental samples. Mycotoxins, which are toxic metabolites of fungi, pose a significant health risk and can contaminate food and feed. Biosensors of this type allow the assessment of mycotoxin contamination in environmental samples such as soil and water, providing a means for rapid screening of contaminated sites, which is an important aspect for ensuring food safety and public health.

In conclusion, in Table 2 the main characteristics of the biosensor systems under consideration.

The use of biosensors to detect organic substances demonstrates a number of significant advantages that determine their effectiveness in various fields, including environmental monitoring and food quality control.

As a general concluding generalization, we can only conclude that the above review material will be very useful to a wide range of medical, engineering, and analytical specialists, as well as scientists who direct their research to the problems indicated in the review. Promising research avenues center on harnessing synthetic biology to engineer highly specific and sensitive microbial responses, synergistically combined with advanced nanomaterials and electrochemical methods for faster, more robust signal generation. Integrating these improved sensing elements within microfluidic platforms and leveraging AI for data analysis from sensor arrays offers a powerful strategy to achieve the required accuracy and efficiency for real-world pollutant monitoring.

It can also be noted that the improvement of bioanalytical systems based on whole microbial cells is a multifaceted process that includes the optimization of microbial characteristics, the integration of advanced materials and technologies, and the application of rigorous analytical methodologies. Despite all the progress in the field of microbial biosensors, there are still key issues that must be addressed to enhance pollutant detection for their broader application. These include enhancing the specificity of sensors against interfering compounds in complex samples and increasing sensitivity to meet stringent regulatory limits. Furthermore, enhancing operational robustness, stability for longer shelf-life, and reducing response times are critical for reliable, real-time field deployment. Successfully tackling these challenges is essential for translating microbial biosensors from promising research concepts into widely adopted analytical tools.

## Figures and Tables

**Table 1:** MPCs of heavy metals in drinking water.

Metal	MPC, mg/L (China)	MPC, mg/L (Europe)
Cd	0.005	0.005
Pb	0.01	0.01
Hg	0.001	0.001
As	0.01	0.01
Cr	0.05	0.025
Zn	1.0	5.0

**Table 2:** The main characteristics of the considered microbial biosensors.

	**Determination** ***of BOD***					
**Biomaterial**	**Type of Electrode/Modification of the Electrode**	**Range of Determined Concentrations, mg O_2_/L**	**Sensor** **Stability, %**	**Detection Mode**	**Testing on Real Samples**	**Reference**
*P. yeei* SPB1	Ferrocene	1.3–360	2.9	Amperometry	-	[61]
*O. polymorpha* and *B. adeninivorans*	Ferrocene	2–140	-	Amperometry	-	[61]
Biofilm	Carbon nanotubes	0.41	5.96	Amperometry	R^2^ = 0.9901 Natural and waste water	[62]
*E. coli* biofilm	Ferrocene	0.87–14	7.69	Amperometry	R^2^ = 0.9862 Freshwater and wastewater	[67]
*Shewanella loihica* PV-4	-	0–435	-	Amperometry	-	[69]
*B. adeninivorans*	Ferrocene–neutral red	0.16–2.7	1.5	Amperometry	R^2^ = 0.9693 Surface waters	[71]
*B. adeninivorans*	Carbon nanotubes and poly(thionine)-NR	0.4–62	3.2	Amperometry	R^2^ = 0.9998 Wastewater	[76]
*Paracoccus yeei* VKM B-3302	Polyvinyl alcohol matrix	0.05–5	7	Amperometry	R^2^ = 0.9990 Natural and waste water	[77]
Active sludge	-	25–500	10	MFC	-	[84]
Electroactive microorganisms	-	10–500	-	MEC	-	[88]
*E. coli* 0157	-	0–27	-	Fluorescence detection	R^2^ = 0.91 for River A and R^2^ = 0.93 for River B	[89]
*Debaryomyces hansenii* VKM Y-2482	Nanostructured electrochemical sensor, ferrocene-methylene blue	2.0–190	3.5	Amperometry	R^2^ > 0.98 Surface waters	[234]
	**Determination of Toxicity**					
**Biomaterial**	**Type of Electrode/Modification of the Electrode**	**IC_50_. mg/L**		**Detection Mode**	**Testing on Real Samples**	**Reference**
*S. cerevisiae* S288C	Glassy carbon (GC) electrode covered with chitosan hydrogel polymer film with boron-doped nanocrystalline diamond (BND). two-mediator system (K_3_[Fe(CN)_6_]. menadione)	10.12 (Cu^2+^) 13.88 (Cd^2+^) 17.06 (Ni^2+^) 34.56 (Pb^2+^) 44.55 (phenol) 34.40 (4-chlorophenol) 16.48 (DCP)		Amperometry	Landfill, electroplanting, and laboratory wastewater	[99]
*G. oxydans* VKM B-1280	Oxygen electrode	16.5 (Fe^3+^) >200 (Cd^2+^) 13.9 (Cr^3+^) 7.2 (Zn^2+^) 12 (Mn^2+^) >200 (TCA) >200 (phenol) >200 (salicylic acid) 2.9 (2,4-dinitrophenol)		Amperometry	Aqueous extracts of samples of industrially produced goods	[98]
	Graphite-paste electrode/ferrocene	7.8 (Fe^3+^) 1.6 (Cd^2+^) 0.8 (Cr^3+^) 2.4 (Zn^2+^) 0.3 (Mn^2+^) 15.7 (TCA) 17.5 (phenol) 19.0 (salicylic acid) 6.8 (2,4-dinitrophenol)		Amperometry		
	MFC (2.6-DCPIP)	1.2 (Cd^2+^) 4.5 (Zn^2+^) 1.6 (Mn^2+^) 24.2 (phenol) 0.9 (2,4-dinitrophenol)		MFC		
*P. yeei* VKM B-3302	Ferrocene	9.9 (Pb^2+^) 18.2 (Cd^2+^) 21.1 (Cu^2+^) 47.5 (Zn^2+^) 9.9 (phenol) 2.1 (p-nitrophenol)		Amperometry	Perfumery and cosmetics samples	[100]
Association of *S. cerevisiae* VKM Y-1173/*P. yeei* VKM B-3302	BSA-NR-CNT/COOH nanocomposite	3.2 (Pb^2+^) 7.6 (Cd^2+^) 8.9 (Cu^2+^) 22.1 (Zn^2+^) 7.5 (phenol) 5 (p-nitrophenol)		Amperometry	Natural waters	[61]
Association of *P. yeei* VKM B-3302/*E. coli* K-802	Ferrocene	7.3 (Pb^2+^) 6.6 (Cd^2+^) 23.8 (Cu^2+^) 2.3 (Zn^2+^) 8.1 (phenol) 29.2 (p-nitrophenol)		Amperometry	Wastewater	[101]
	**Determination** ***of Heavy Metals***					
**Biomaterial**	**Heavy Metal**	**Range of Determined Concentrations, mg/L**	**Limit of Detection, mg/L**	**Detection Mode**	**Testing on Real Samples**	**Reference**
*E. coli* K12-PMP-*luxCDABE*-△*cysI*	Cd (II)	0.005–2	0.005	Fluorescence detection	Drinking water	[125]
GFP *Bacillus megaterium* VR1/SiNa/LUDOX	Cd	0–10	1.42 × 10^−4^	Fluorescence detection	-	[124]
	Cu	0–20	3.16 × 10^−4^			
	Zn	0–100	2.42 × 10^−4^			
Dual-colored bacterial biosensor (CadR-regulated vioABE and a MerR-regulated VioC expression module)	Cd	5.5 × 10^−4^–4.5	5.5 × 10^−4^	Colorimetric	Seawater	[119]
	Pb	5.06 × 10^−3^–41.4	5.06 × 10^−3^			
	Hg	7.42 × 10^−4^–9.4 × 10^−4^	7.42 × 10^−4^			
	**Determination of Surfactants**					
**Biomaterial**	**Range of Determined Concentrations, mg/L**		**Response Measurement Time, min**	**Detection Mode**	**Testing on Real Samples**	**Reference**
*Herbaspirillum lusitanum* P6–12	0.01–0.1		1–5	Electric polarizability detection	-	[139]
*Comamonas testosteroni* TI	0.25–0.5		12–15	Amperometry	-	[235]
*Pseudomonas rathonis*	0.25–0.75		1.7–2.5	Amperometry	-	[236]
Biosensor based on whole cell transcription factor	0.48–62.5		-	Fluorescence detection	Sewage (RSD = 2.4%) and pond (RSD = 2.8%) water	[136]
*E. coli*	1.7		1	Fluorescence detection	Tap water, river water, and drinking water	[45]
	**Determination of Phenols**					
**Biomaterial**	**Type of Electrode/Modification of the Electrode**	**Detectable Compound**	**Range of Determined Concentrations, M**	**Detection Mode**	**Testing on Real Samples**	**Reference**
*Staphylococcus aureus*	Copper electrode modified with a poly-caprolactone film	Phenol	0.01–0.05	Square-wave voltammetry	-	[146]
*Pseudomonas putida* BS394 (PBS216) (adapted for phenol)	BSA-FC/CNT Redox-Active Biocompatible Composite Polymer “Bovine Serum Albumin–Ferrocene–Carbon Nanotubes”.	Phenol, 2,4-dinitrophenol	1.063 × 10^−8^–0.002 (phenol)	Amperometry	River water	[155]
*Pseudomonas* SP. (GSN23) (adapted for phenol)	IDEs-MWCNTs	Phenol ƿ-Nitrophenol Bisphenol-A 4-chlorophenol 2,3,6-trichlorophenol 2,4,6-trichlorophenol	1 × 10^−5^–0.003187 (phenol)	Conductometry	-	[156]
pUC57-QS-DSF-F42 L/*E coli* DH5α	-	Phenol, p-nitrophenol	1 × 10^−7^–5 × 10^−4^ (phenol)	Fluorescence detection	-	[157]
*E. coli*-Tyrosinase	Glassy carbon (GCE) electrode	Bisphenol	1 × 10^−11^–1 × 10^−7^	Amperometry	Tea and juice	[161]
*E. coli* BL21	Nanoporous gold (NPG)/GCE	Catechol	1 × 10^−6^–5 × 10^−4^	Differential pulse voltammetry	Synthetic wastewater	[43]
	**Determination of Pesticides**					
**Biomaterial**	**Detectable Pesticide**	**Range of Determined Concentrations, µM**	**Limit of Detection, nM**	**Detection Mode**	**Testing on Real Samples**	**Reference**
*E. coli* BL21/pPNP-LacZ	Methylparation	0.04–38	20	Colorimetric	R^2^ = 0.997 Soil	[174]
*S. cerevisae* EBY100	Parathion	0.017–34.4	12.8	Visible spectrophotometry	Tap water (RSD = 2.7%), seawater (RSD = 5.9%), and sewage (RSD = 3.2%)	[180]
	Paraoxon	0.018–36.3	0.49		Tap water (RSD = 4.5%), seawater (RSD = 2.7%), and sewage (RSD = 6.3%)	
*E. coli* BL21	Paraoxon	-	2000	Fluorescence detection	-	[181]
*E. coli* PC16	Chlorpyrifos	-	5	Fluorescence detection	Seawater, wastewater	[182]
*A. variabilis* SA-1	Atrazine	0–1.31	70	Photoelectrochemical (amperometry)	-	[183]
*Leptolyngbia* sp.	Atrazine	0.1–1.2	14	Photoelectrochemical (amperometry)	Tap water	[184]
*Chlamydomonas reinhardtii*	Nanocapsulated atrazine	0.010–0.150	0.004	Fluorescence detection	Tap water	[44]
Microorganisms of anaerobic sludge	Atrazine	0.2–1	200	MFC	-	[185]
*Agrobacterium tumefaciens* NTL4	2,4-dichlorophenoxyacetic acid	0–100	1560	Fluorescence detection	Environmental water	[186]
*S. cerevisiae* BY4741	Tebuconazole	-	20	Bioluminescence detection	Environmental water	[187]
*E. coli*	Dicamba	0–4500	-	Cyclic voltammetry	Environmental water	[188]
*E. coli* MG1655	3,5-dichlorophenol	20–1500	-	Raman spectroscopy	-	[194]
	**Determination of Other Organic Pollutants**					
**Biomaterial**	**Detectable Compound**	**Range of Determined Concentrations, µg/L**	**Limit of Detection, µM**	**Detection Mode**	**Testing on Real Samples**	**Reference**
*E. coli DH5α*	Benzene. Toluene, and xylene	-	0.24	Bioluminescence detection	Seawater	[237]
*B. sartisoli* RP007	Naphthalene and phenanthrene	-	0.17		Seawater	
*Acinetobacter* ADPWH_Nah	Naphthalene	-	0.01	Colorimetric	Groundwater and soil	[238]
*E. coli*/pMTLacZ.	Tetracyclines	75–10,000	0.011 (chlorotetracycline) 0.012 (deoxytetracycline) 0.013 (tetracycline) 0.037 (minocycline) 0.039 (methacycline)	Bioluminescence detection	Environmental water	[239]

## Data Availability

Data are contained within the article.

## Abbreviations

The following abbreviations are used in this manuscript:

EIS	Electrochemical impedance spectroscopy
MFC	Microbial fuel cell
AI	Artificial intelligence
IoTs	Internet of Things
BESs	Bioelectrochemical systems
QbD	Quality by Design
BOD	Biological oxygen demand
EAMs	Electroactive microorganisms
EABs	Electroactive biofilms
SWCNTs	Single-walled carbon nanotubes
MEC	Microbial electrolytic cell
AAS	Atomic absorption spectroscopy
OES	Optical emission spectroscopy
SDS	Sodium dodecyl sulfate
MPC	Maximum permissible concentration
HPLC	High-performance liquid chromatography
MWCNTs	Multi-walled carbon nanotubes
SEM	Scanning electron microscopy
BPA	Bisphenol A
OPs	Organophosphate pesticides
MP	Methylparation
p-NP	p-Nitrophenol
IPTG	Isopropyl-β-D-thiogalactopyranoside
QS	Quorum sensing
AHL	N-acylhomoserinlactone
AChE	Acetylcholinesterase
FGE	Formylglycine generating enzyme
OPH	Organophosphate hydrolase
BPV	Biological photovoltaic cell
Box	Bilirubinoxidase
2,4-D	2,4-dichlorophenoxyacetic acid
TEB	Tebuconazole
RSD	Relative standard deviation
